# Multitarget-Directed Ligands for Alzheimer’s Disease: Recent Novel MTDLs and Mechanistic Insights

**DOI:** 10.3390/ph18111685

**Published:** 2025-11-07

**Authors:** Mohammed Almaghrabi

**Affiliations:** Department of Pharmacognosy and Pharmaceutical Chemistry, College of Pharmacy, Taibah University, Al-Madinah Al-Munawarah 30001, Saudi Arabia; mhmaghrabi@taibahu.edu.sa

**Keywords:** Alzheimer’s disease (AD), multitarget-directed ligands, knowledge-based approaches, fused pharmacophores, linked pharmacophores

## Abstract

Alzheimer’s disease (AD) is the most prevalent neurodegenerative disease, affecting millions of people and challenging the public health framework globally. While the definitive cause of AD remains unclear, researchers are concentrating their efforts on several prominent theories. Currently, there are very few FDA-approved medications for AD, and these primarily alleviate symptoms rather than alter the disease’s progression. In response, research efforts focus on developing new medicines that address the complex nature of AD through multi-targeted approaches. Multitarget-directed ligands (MTDLs) are a promising treatment strategy for AD, despite the significant challenges they pose. This review examines recent advancements in designing prospective targeted MTDLs to combat AD, with a focus on categorizing the lead generation process and investigating the integration methods of key pharmacophores within the 2024–2025 timeframe. The review highlights numerous examples of novel MTDLs that address various AD hallmarks, demonstrating their broad therapeutic potential. These targets and activities include cholinesterase (AChE and/or BuChE) inhibition, monoamine oxidase (MAO-A and/or MAO-B) inhibition, antioxidant activity, amyloid-beta (Aβ) aggregation inhibition, tau protein aggregation inhibition, glycogen synthase kinase 3β (GSK-3β) inhibition, calcium channel blockade, anti-inflammatory activity, and other hallmarks.

## 1. Introduction

Neurodegenerative diseases (NDDs) constitute a major global health challenge, encompassing a group of conditions characterized by progressive loss of neurons in the brain and central nervous system. These complex and multifactorial diseases include disorders such as Parkinson’s disease, Huntington’s disease, amyotrophic lateral sclerosis (ALS), and multiple sclerosis (MS) [1]. Alzheimer’s disease (AD) is among the most prevalent NDDs and the primary cause of dementia. Dementia is a syndrome characterized by a decline in cognitive function, including memory loss, communication difficulties, and personality changes, that interferes with daily life. AD, specifically, is a complex, gradually progressive neurological disorder associated with neuronal damage that leads to a decline in brain functions [2,3].

The global prevalence of AD poses a massive social and economic burden. Approximately 55 million individuals worldwide are affected by AD. This number is expected to increase dramatically by 2050, with a pronounced 68% increase in dementia cases forecasted for low- and middle-income countries. In 2019, AD and other dementias were responsible for an estimated 1.6 million deaths globally. This alarming trend drastically escalates the need for medical care and other supportive services, resulting in a considerable financial impact [4,5,6].

Several key risk factors are associated with AD, with advanced age being the most critical factor. Other factors include genetic elements, such as mutations in APP and PSEN1/2, environmental factors, immune system dysfunction, vascular disease, traumatic brain injury (TBI), infection, metal exposure, and poor diet [7]. AD diagnosis can be challenging; patients with AD usually have mixed symptoms, and it is rare for a patient to have apparent AD symptoms. Therefore, the definitive diagnosis of the condition currently relies on conducting a neuropathological examination during an autopsy [8]. However, an accurate diagnosis of AD can be made by considering general clinical and pathological factors, as well as evaluating several in vivo biomarkers and memory tests [9]. AD staging is categorized into two distinct dimensions. Clinical staging employs a six-stage numerical system (0–6) to classify the severity of cognitive and functional decline, spanning from Stage 1 (asymptomatic individuals with only biomarker evidence) to Stages 4–6, which signify progressive loss of independence and worsening functional impairment. In contrast, biological staging, categorized as A–D, defines the severity of AD only by its underlying biology, employing core biomarkers (in A and T categories), which reflect the stereotypical progression from Stage A to Stage D [10].

Looking ahead, AD care is anticipated to remain a significant public health concern. Addressing future concerns about AD will require increasing knowledge and research through various methods, which may be advantageous, including the identification and management of risk factors, as well as the development of new techniques for early diagnosis and effective treatment [6].

## 2. Alzheimer’s Disease Pathogenesis

Considerable advancements in understanding AD have occurred over the past few decades, yet the underlying cause remains indefinite, with several theories attempting to elucidate its pathogenesis [11]. The two most extensively studied and influential theories are the cholinergic hypothesis and amyloid-beta (Aβ) dysregulation. The cholinergic hypothesis arose from the discovery of a deficit in the enzyme choline acetyltransferase (ChAT), which is responsible for synthesizing acetylcholine (Ach). This deficit leads to the degeneration of cholinergic neurons in the basal forebrain, followed by a loss of cholinergic neurotransmission in the cerebral cortex and other brain areas, which is strongly linked to impaired cognitive function [12].

The Aβ cascade theory proposes that memory and cognitive impairment result from the excessive production and accumulation of Aβ peptides, which form insoluble senile plaques outside neurons. The Aβ peptide is generated from the amyloid precursor protein (APP) through a process regulated by the β-secretase enzyme (BACE1). This enzyme catalyzes the cleavage of APP, a rate-limiting step in AD pathogenesis that produces a 99-amino-acid fragment called C99. The resulting fragment is then further processed by γ-secretase, which cuts the C99 at multiple sites to generate insoluble, neurotoxic Aβ peptides, predominantly Aβ40 and Aβ42 [13,14]. These Aβ peptides tend to form Aβ monomers, which aggregate into insoluble oligomers and ultimately into amyloid plaques. These Aβ aggregates are linked to synaptic dysfunction through neurotoxic mechanisms, as they alter the mitochondrial structure of neuronal cells and disrupt the balance of calcium. This disruption results in decreased ATP production and an imbalance in mitochondrial fission and fusion. Consequently, Aβ plaques stimulate the production of free radicals, which in turn affect Ca^2+^ homeostasis and lead to oxidative stress [13,14,15].

Oxidative stress itself is a major contributor to AD pathogenesis. The brain is highly susceptible to oxidative damage due to its high oxygen consumption and metabolic rate, combined with neurons that are rich in polyunsaturated fatty acids. The primary source of reactive oxygen species (ROS) is the mitochondrial electron transport chain during oxidative phosphorylation. Oxidative stress damages critical neuronal components, including lipids, proteins, and nucleic acids, which elevate AD biomarkers such as protein carbonyls and malondialdehyde [16,17].

Neurofibrillary tangles (NFTs) appear as insoluble clumps within neurons. Their formation is associated with the accumulation and misfolding of Aβ, along with oxidative stress. These factors promote excessive hyperphosphorylation of tau protein, leading to its aggregation into oligomers and eventually into NFTs. The phosphorylation process is controlled by various kinases, particularly glycogen synthase kinase 3 (GSK-3β) and cyclin-dependent kinase 5 (CDK5), which are frequently activated by extracellular Aβ. The creation of NFTs disrupts communication between neurons and impairs signal transmission [18,19].

**Figure 1:**
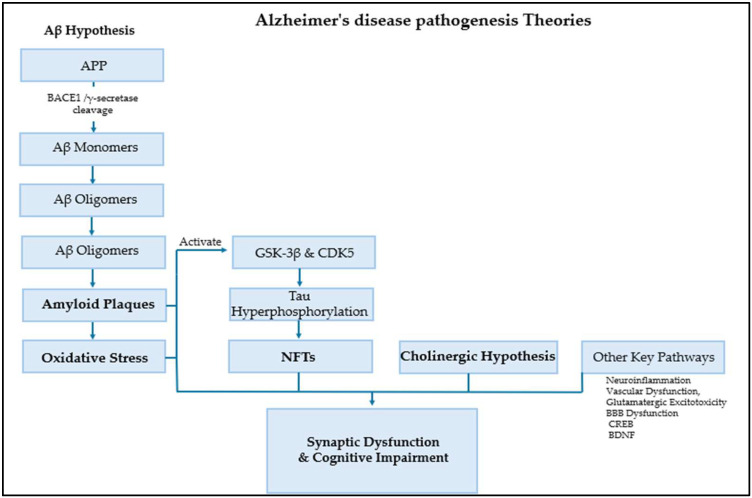
AD pathogenesis theories.

AD pathogenesis is driven by a complex interaction of multiple fundamental pathways and numerous contributing factors. Established theories shed light on the amyloid cascade, in which the accumulation of neurotoxic Aβ plaques initiates synaptic damage and oxidative stress, alongside tau hyperphosphorylation and formation of NFTs. However, many other important factors play a critical role in the pathogenesis of AD, including neuroinflammation, calcium signaling dysregulation, glutamatergic excitotoxicity, vascular dysfunction, monoamine oxidase MAO, and blood–brain barrier (BBB) dysfunction [20,21,22,23]. Additionally, Aβ toxicity suppresses the cAMP response element-binding protein (CREB), thereby disrupting neurotrophic systems, such as brain-derived neurotrophic factor (BDNF) and its receptor, TrkB. This disruption directly contributes to the synaptic dysfunction and cognitive decline characteristic of AD, Figure 1 [24].

## 3. Alzheimer’s Disease Treatment

Intensified research into AD has increased recently, but current treatments remain limited, and the success rates of treatments are very low. The U.S. Food and Drug Administration (FDA) has approved only a limited number of medications for treating the disease. Critically, these agents primarily manage symptoms rather than altering the disease’s underlying pathological course [25,26].

The approved drugs can be categorized into a few classes based on their mechanism of action. The first class is acetylcholinesterase (AChE) inhibitors, which include donepezil, galantamine, and rivastigmine. AChE inhibitors work by blocking the breakdown of ACh, thereby extending its duration of action and increasing its concentration in the cerebral cortex [27]. Tacrine was the first AChE inhibitor to be introduced in clinical settings of AD treatment. However, due to its potential for causing liver damage, it was removed from the market [28]. Later generations of AChEIs provided safer alternatives; for instance, donepezil, a second-generation AChE inhibitor, is a primary AD treatment known for being highly selective, reversible, and acting as both a mixed competitive and non-competitive inhibitor [29]. Galantamine acts as a selective, competitive, and reversible AChE inhibitor, and its action also involves modulating nicotinic acetylcholine receptors, thereby potentially augmenting its cognitive-enhancing properties [30]. Finally, rivastigmine is another AChE inhibitor commonly used for AD. Rivastigmine acts differently from donepezil and galantamine by inhibiting AChE and butyrylcholinesterase (BuChE) in the brain [30].

The second class consists of a single N-methyl-D-aspartate receptor (NMDAR) antagonist, memantine. Memantine acts as a non-competitive NMDA receptor blocker, thereby preventing excess calcium from entering the brain to reduce symptoms associated with AD. Notably, these medications can be used separately or in combination to treat the symptoms of AD. It appears that their different mechanisms of action create a synergistic effect [31].

A groundbreaking advancement in AD treatment has emerged with the recent approval of anti-amyloid monoclonal antibodies. Lecanemab and donanemab represent a turning point in AD treatment and research by targeting a core pathology of the disease. These new agents are classified as anti-amyloid antibodies, functioning as disease-modifying therapies that actively eliminate amyloid deposits, unlike earlier antibodies that primarily aimed to prevent accumulation. Clinical trials have shown that both lecanemab and donanemab reduce disease progression by 25% to 30% over the course of their respective trials, which lasted approximately 18 months. However, their mechanism is associated with a primary safety risk, known as Amyloid-Related Imaging Abnormality (ARIA). ARIA, comprising edema/effusion (ARIA-E) and hemorrhage/siderosis (ARIA-H), originates from an inflammatory response. This reaction occurs when the antibody collides with amyloid in the blood vessel walls [32,33].

## 4. Alzheimer’s Disease Multi-Target Treatment

Given the complex and multifaceted nature of AD, multi-target approaches are gaining growing interest as a potential treatment strategy. The limitation of current monotherapy of AD (one molecule, one target, one disease) urges researchers to adopt alternative strategies with the hope of treating multifactorial diseases such as AD. These alternative strategies include combination therapy, “drug cocktails,” where two or more drugs are combined to address multiple targets [34,35].

Evidence suggests that combination therapies can achieve superior outcomes in AD treatment. Parallel to effective regimens for therapies for cancer and HIV, the synergistic approach of medications targeting multiple areas for AD enables the simultaneous tackling of more than one symptom [36]. Despite the advantages of a combined treatment approach, such as synergistic results and minimal dosages, there are several limitations, for instance, poor patient compliance, increased risk of drug–drug interaction, toxicity that restricts dosage, and complicated pharmacokinetic and pharmacodynamic profiles [37].

Developing medications that function as multi-target inhibitors, thereby shifting the paradigm from one target, one disease, to working on multiple targets, became a key objective in treating AD’s multifactorial nature [3,38]. The promising concept of a single molecule with numerous functions is referred to by various terms in the literature, including dual mechanism, bifunctional, hybrid compounds, multi-functional compounds, and multi-directed ligands [39]. While molecular hybridization is a broad strategy that combines two or more pharmacophores into a single molecule, this review will focus specifically on multitarget-directed ligands (MTDLs), which represent a more rational design approach. In essence, MTDLs are a polypharmacological strategy that incorporates different pharmacophores into a single molecule to target multiple biological pathways [40].

The general concept of MTDLs was not unfamiliar in the drug development process, as many medications have acted through multiple molecular mechanisms for decades. A prime example is the discovery of aspirin by chance [41]. Until 2004, Morphy et al. and their team suggested using the term “Designed Multiple Ligands” (DMLs) to describe a rationally designed single molecule with a multi-biological profile, addressing a particular disease [38]. Over time, this term has developed to become the predominant and widely accepted term, the MTDLs. The primary objectives of MTDLs’ approach are to offer enhanced therapeutic benefits, increase efficacy, improve safety, decrease drug resistance, and provide more predictable pharmacokinetic and pharmacodynamic profiles [42]. Consequently, the discovery and development of MTDLs have been a focal point for the past two decades, and the rational design of MTDLs has become more sophisticated and grounded in scientific methods. This development process includes several complex key steps and strategies [43].

A rational combination of targets is an initial step in the MTDLs process, which involves selecting a target combination that yields enhanced therapeutic effects through synergistic interactions. It is a strategy based on the disease’s multifaceted nature and the potential for achieving synergistic outcomes. This step is typically guided by clinical observation, phenotypic screening, and in silico techniques, followed by experimental validation [43,44]. The core design concept for an MTDL agent is to retain the original activity of a pharmacophore while incorporating a second one. Consequently, the MTDL pharmacophore should preserve the primary interaction while also being compatible with the second target. The lead generation step for MTDLs includes two main approaches: knowledge-based approaches and screening approaches [42,45].

Knowledge-based or pharmacophore-based approaches are the most common methodologies for designing new MTDL agents. Generally, this process combines two or more well-known pharmacophores into one single entity molecule, “design in”, to integrate the activities of these pharmacophores. The classification of resulting MTDLs relies on the types of pharmacophore integration, which is categorized into linked, fused, and merged pharmacophores [46,47].

In the merged pharmacophore approach, the functional groups of two or more lead compounds are integrated into a single compound, requiring that the binding sites of both lead compounds are similar. The highest level of pharmacophore overlaps occurs when the pharmacophores of various molecules are closely integrated by utilizing the common structural characteristics of the original compounds. This process also involves identifying the typical characteristics of both lead compounds or ligands, as well as key pharmacophore features such as ionic bonds and hydrophobic interactions, and evaluating the binding sites. The goal is to achieve a smaller molecule and optimize the physicochemical properties of the new compound [43,48].

The fused pharmacophore approach involves directly fusing two key pharmacophores, a method employed when the two targets have large binding sites. Without using a linker, this method partially overlaps the pharmacophores while considering important groups that bind to the exposed solvent area [42,49].

Finally, the linked pharmacophores approach is divided into cleavable and non-cleavable types; in this method, a linker is used to connect the two pharmacophores of two lead compounds. This strategy is typically applied when small binding regions are deep in the protein. Non-cleavable MTDLs feature a metabolically stable linker and are designed to bind in vivo to two distinct targets as an integral molecule. The cleavable MTDLs are designed to hydrolyze in vivo, allowing the separate pharmacophores to bind to their targets individually [43,50].

In contrast, screening approaches depend on finding a promising starting ligand by testing compound libraries or known drugs. This approach has two main categories: focused screening, which is screening compounds known to be effective against one target for activity against another target. The other is diversity-based screening, also known as high-throughput screening (HTS), which involves screening large and diverse compound libraries for activity against a single target and then verifying hits against a second target [51,52]. Following lead identification, the lead optimization step requires balancing MTDL activities across multiple targets to ensure maximum clinical relevance with an optimal ratio of affinities for all targets [37]. Synthesizing MTDLs is typically a complex process that poses more significant challenges than that of traditional single-target medications. The efficiency of MTDLs depends on the nature of the template ligands, the chosen linkage site, the linker length, and the use of modern synthetic methodologies [42].

Several challenges and considerations must be addressed during the generation of MTDLs, including physicochemical and pharmacokinetic (PK) challenges, as multi-target drugs tend to be larger and more lipophilic than those targeting a single protein. Moreover, template ligands should hold comparable physical and chemical properties and similar biological activity. Also, achieving selectivity over closely related target subtypes presents another major challenge. Other critical considerations include fusion or conjugation approaches for MTDLs when the targets are dissimilar, and managing the activity of new MTDL metabolites, stereochemistry, and the different types of pharmacophore activities within one molecule [45,53].

## 5. Most Recent Novel MTDLs Developed via Pharmacophore Design Strategy

### 5.1. Linked Pharmacophore Strategies

**Figure 2:**
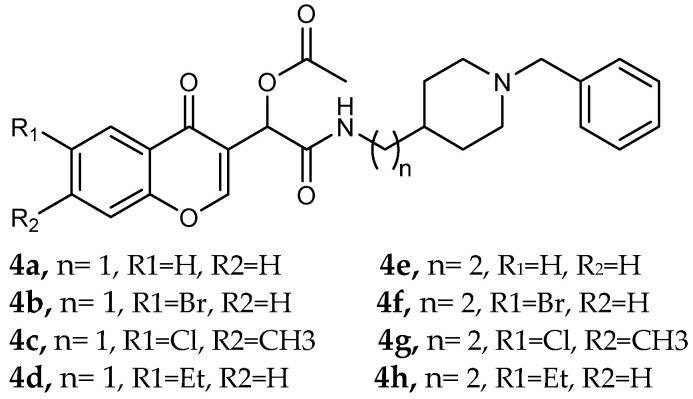
Chromone–donepezil hybrids.

Simakov et al. [54] investigated eight novel multi-target-directed ligands, compounds **4a** through **4h**, which combine chromone and donepezil moieties as potential AD treatments (Figure 2). The design of these MTDLs was based on a knowledge-based approach, employing a non-cleavable linked-pharmacophore strategy to connect the two pharmacophores with a linker.

In their evaluation, all tested compounds effectively activated the Nrf2/ARE pathway. The researchers used CD values—the concentration needed to double the reporter activity—to measure potency, with tert-butylhydroquinone (tBHQ, CD = 0.6 µM) as a reference. Compounds **4b, 4c, 4f**, and **4h** demonstrated higher activity than tBHQ, evidenced by their lower CD values of 0.4 µM for **4b** and **4f**, 0.3 µM for **4c**, and 0.5 µM for **4h** (Table 1). Compounds **4d** and **4g** showed activity similar to tBHQ (CD = 0.7 µM), while **4a** and **4e** were less potent (CD = 1.0 µM).

Regarding Nrf2 downstream gene activation, the compounds effectively induced the expression of NQO1. Specifically, compounds **4a, 4c, 4d**, and **4g** showed substantial activation of the NQO1 pathway, matching or exceeding the effect of tBHQ. The expression of HO1 was also significantly induced by compounds **4b**, **4c**, **4d**, and **4g**. Furthermore, compound **4d** was identified as the only analog with significant direct antioxidant activity in SH-SY5Y cells, as shown in Table 1.

The study also assessed calcium channel blockade for compounds **4a–l** using nimodipine as a reference. This essay was conducted in SH-SY5Y neuroblastoma cells, where fluorescence changes were recorded. From the twelve tested compounds, three were inactive, while the active ones showed a range of inhibitory effects from 3% for compound **4c** to 19% for compound **4f** (Table 1).

Zhao et al. [55] studied the potential of the natural product, usnic acid (UA), as a novel therapeutic agent for AD. The goal was to design, synthesize, and characterize two usnic acid derivatives, **1** and **2**, by introducing a dimethylamine Schiff base-containing side chain moiety into the “toxic triketone” portion of UA to enhance anti-AD effects and reduce toxicity. Researchers employed a non-cleavable linked pharmacophore approach, which combines two distinct moieties via a linker.

Using Ellman’s method, the inhibitory activity of compounds **1** and **2** was considerably more vigorous against AChE (from electric eel) and BuChE (from equine serum) when compared to UA. Compounds **1** and **2** demonstrated significant inhibition of AChE, with IC50 values of 0.713 μM and 0.143 μM, respectively, while the UAs were greater than 100 μM. The IC50 values for BuChE inhibition were 5.934 µM for compound **1** and 0.356 µM for compound **2**, compared to values exceeding 100 µM for UA. The positive control, tacrine, had IC50 values of 0.0145 μM for AChE and 0.003 μM for BuChE. The enhanced inhibitory activity supported the design approach of incorporating tertiary amine groups into the UA structure. Compound **2** exhibited enhanced inhibition of cholinesterase activity, likely due to its extended side chain, which enables it to penetrate further into the active site.

Antioxidant activity was assessed using ·OH scavenging ability via the Fenton reaction. The tertiary amine and hydroxyl groups contributed to antioxidant activities, with compounds **1** and **2** showing ·OH scavenging capacities similar to UA. The CV results indicated similar ·O2−-scavenging capabilities to UA, and in vivo C. elegans experiments confirmed their effective antioxidant properties and substantial ROS scavenging activity.

For metal chelation, compound **2** formed a single crystal with ZnCl2, yielding a Zn(II) complex (2a) where UA and the tertiary amine act as mixed ligands. X-ray diffraction revealed that the Zn(II) ion is coordinated to six atoms, including oxygen from UA and nitrogen from the tertiary amine. Researchers discovered that compound **2** undergoes substantial breakdown in the presence of Zn(II) ions, and following hydrolysis, the resulting compounds can bind to zinc to form complex 2a. The “triketone” functional group was crucial in coordinating Zn(II) ions, suggesting that this modification could help regulate metal balance and act as a detoxifier (Table 2).

Abd El-Mageed et al. and their coworkers [56] focused on creating novel MTDLs of coumarin derivatives capable of suppressing the main AD hallmarks, including tau protein aggregation, Aβ aggregation, and the inhibition of GSK-3β, AChE, and/or BuChE (Table 3). The study featured two series: compounds **6a**–**i** utilized a coumarin scaffold as a planar heteroaromatic head to interact with the catalytically active site (CAS) of AChE, thereby preventing the stabilization and hydrolysis of acetylcholine (ACh), while series **9a**–**g** incorporated an amide scaffold with a nitrile group. The second series was specifically designed to enhance ACh stability and improve binding capacity.

The MTDL strategy employed a non-cleavable linked pharmacophore approach. In the first series, a hydrophobic tail was incorporated to interact with AChE’s CAS, while the second series used an amide moiety and nitrile group to improve stability and binding.

Compounds **6c** and **6h** showed significant inhibition of human acetylcholinesterase (hAChE), with IC50 values of 28.88 nM and 26.03 nM, respectively, compared to donepezil (IC50 = 31.54 nM). Studies of kinetic analysis and docking revealed that compound **6h** exhibited a dual-site inhibitory effect on hAChE; it may bind to both the CAS and PAS of the enzyme. This dual binding ability elevates ACh levels and may postpone AD progression by inhibiting Aβ aggregation. Compounds **6c** and **6h** confirmed valuable inhibitory activity against hBuChE, with IC50 values of 103.90 nM and 90.09 nM, respectively. Compound **6h** showed higher potency than rivastigmine (IC50 = 107.40 nM). For GSK-3β inhibitory activity, compounds **6c** and **6h** inhibited GSK-3β in the nanomolar range with an IC50 value of 51.42 nM and 26.91 nM, respectively. These compounds were found to be 4 to 8 times more efficient than donepezil, with an IC50 value of 219.10 nM. Compounds **6c** and **6h**, when compared to donepezil, were found to have significant inhibition of tau protein aggregation, with IC50 values of 31.22 μM and 56.31 μM, respectively, while donepezil was found to have an IC50 value of 120.1 μM. When compared to donepezil, compounds **6c** and **6h** also exhibited more significant inhibition of Aβ aggregation than donepezil (IC50 = 75.31 μM), with IC50 values of 22.45 μM and 35.04 μM, respectively, as shown in Table 3.

Negi et al. study [57] designed, synthesized, and evaluated novel MTDL inhibitors of AChE and MAO-A & B (Table 4). The goal of the research was to combine two distinct scaffolds, piperidine and isatin, to yield compounds with dual inhibitory activity. These scaffolds were chosen as key building blocks in drug design due to their favorable pharmacokinetic properties, including BBB permeability and ease of structural modification. The design strategy leveraged the known AChE inhibition of benzylpiperidine derivatives and the MAO inhibitory properties of isatin. Twelve novel benzylpiperidine–isatin hybrids (compounds **4**–**15**) were developed using a non-cleavable linked pharmacophore approach, connecting the two active scaffolds through flexible –NH–CH2–CO–NH–N=C linkers.

In vitro evaluation using a rat liver mitochondrial homogenate revealed that most compounds (**5**–**10** and **12**–**15**) exhibited considerable MAO-A inhibitory activity, with IC50 values ranging from the nanomolar to the micromolar range and a general selectivity for MAO-A over MAO-B. Compound **15** was identified as the most potent MAO-A inhibitor (IC50 = 0.108 ± 0.004 µM). For MAO-B inhibition, activity ranged widely, with compound **4** being the most potent (IC50 = 0.057 ± 0.001 µM). Only compounds **4** and **11** showed selectivity towards MAO-B, with compound **4** exhibiting the highest selectivity index (3.85).

All compounds proved moderate to potent AChE inhibition, with IC50 values ranging from nanomolar to micromolar levels. Compound **15** was the most potent AChE inhibitor with an IC50 of 0.034 ± 0.002 µM, outperforming donepezil (IC50 = 0.084 ± 0.002 µM).

Regarding the DPPH antioxidant activity assay, all compounds (**4**–**15**) exhibited acceptable antioxidant effects. Compared to ascorbic acid, compounds **14** and **15** demonstrated higher antioxidant activity, whereas compound **4** displayed antioxidant capability that was almost on par with that of ascorbic acid (Table 4).

Shaaban et al. [58] emphasize the development of novel hybrid compounds with dual inhibitory activities against ChEs and MAOs, as detailed in Table 5. The design strategy incorporates naturally derived cinnamic acid and its derivatives, known for their neuroprotective effects, with other natural product-derived analogues, such as eugenol, coumarin, and isatin, which exhibit MAO inhibitory and antioxidant effects. A 1,2,3-triazole was chosen as a linker to connect these pharmacophores to enhance anti-cholinesterase activity, decrease toxicity, and interfere with Aβ1–42 aggregation. This approach resulted in a non-cleavable linked-pharmacophore design for the eighteen synthesized compounds.

All synthesized compounds showed a range of inhibitory effects from moderate to potent. The potent inhibition of AChE by compound **16** (IC50 = 4.59 μM) is attributed to the incorporation of 3,4-dimethoxycinnamamide, which is linked to a sesamol moiety via a triazole ring. Compound **18** also showed significant activity (IC50 = 6.42 μM) due to the cinnamamide linked to an isatin moiety via a triazole ring, while compound **14** exhibited strong activity (IC50 = 7.51 μM) due to the linker of 3,4-dimethoxycinnamamide to a vanillin moiety via a triazole ring. All previous compounds outperform the well-known AD treatment, donepezil (IC50 6.01 μM). Compounds with a ferulic acid structure displayed no AChE inhibition at concentrations up to 100 μM, with the notable exception of hybrid **13**, which exhibited moderate activity. For BuChE inhibitory activity, most of the hybrids showed IC50 values in the micromolar range, suggesting potent to moderate inhibition. The compounds **16** and **21** exhibited the most significant inhibitory effects, with IC50 values of 13.24 and 13.53 μM, respectively. The activity of compound **16** is associated with a 3,4-dimethoxycinnamic acid framework that is attached to sesamol via a triazole bridge. Compound **21** features a cinnamic acid unit attached to a purine framework via a triazole linkage. Regarding the MAO-A and MAO-B inhibitory activity, compound **16** exhibited the greatest inhibitory potency against MAO-A, with an IC50 of 30.78 μM, and against MAO-B, with an IC50 of 32.02 μM. Additionally, compound **16** exhibited a selective affinity for both MAO-A and MAO-B enzymes, which was attributed to the dimethoxycinnamamide moiety linked to sesamol (Table 5).

The work of Dias et al. [59] describes the design and evaluation of novel MTDLs, specifically rivastigmine–melatonin derivatives, as shown in Table 6. The hybrid compounds were designed by conjugating rivastigmine with melatonin analogues, specifically indole derivatives, through the hydroxyl substituent. This strategy aimed to merge the cholinesterase inhibition of rivastigmine with the antioxidant and metal-chelating properties of melatonin/indole derivatives. The study included a series of nine compounds (**5a1**–**3, 5b1**–**3, 5c1**–**3**); two compounds (**5a2** and **5a3**) were recognized as the most promising MTDL agents. The paper employs a hybridization or conjugation strategy for two distinct pharmacophores joined via a linker, forming a non-cleavable linked-pharmacophore mode.

Compound **5a3** exhibited effective chelating properties for Fe (III) (pFe = 17.0) and Cu (II) (pCu = 9.0), and moderate chelating ability for Zn (II) (pZn = 6.3). Complexation models indicate that Cu (II) prefers N−, N-coordination in a basic environment, resulting in the displacement of both imidic and indolic protons, ultimately yielding red/purple 1:2 Cu/L complexes with a N4-coordination, as verified by EPR and substantiated by DFT calculations. For Fe (III), complexation prefers phenolic coordination, with a probable binding interaction with the adjacent indole amine. The novel compounds presented moderate to good inhibitory potential against Aβ aggregation, ranging from 19.9 to 55.5%. The presence and position of the hydroxyl substituent group within the indole pharmacophore were crucial; compounds **5a3**, **5b3**, and **5c3**, which have an ortho-hydroxyl group relative to the indole amine, exhibited significant activity, ranging from 47.8% to 55.5%. The compounds **5a2**–**3**, **5b2**–**3**, and **5c2**–**3** were more effective at preventing the Cu (II)-triggered formation of Aβ aggregates (45.7–83.7%) than the self-initiated aggregation process. Compounds **5c3** and **5a3** displayed significant efficacy, with 83.7% and 73.5%, respectively. The ability of these hybrids to bind Cu(II) through chelation likely enhances their inhibitory effect on Aβ aggregation triggered by Cu(II) and their potential for intercalating between Aβ fibril beta-sheets.

Regarding MAO I inhibitory activity using in vitro human recombinant MAO isoforms (hMAO-A and hMAO-B), compound **5a2** exhibited the most potent inhibitory activity of hMAO, with IC50 values of 6.66 μM for hMAO-A and 3.85 μM for hMAO-B. In addition, compound **5a2** was more selective towards hMAO-B, with a selectivity index = 1.7. The absence or presence of a hydroxyl group at position 7 typically leads to weak hMAO inhibition compared to **5a3**. Other compounds, like **5c2** and **5c3**, also presented notable hMAO-B inhibition, with 44% and 39%, respectively (Table 6).

Asghar et al. and their coworkers [60] evaluated the potential inhibitory effects of twelve tryptamine derivatives against key AD targets (Table 7). Tryptamine is a promising molecular scaffold for developing new MTDL leads for AD, with derivatives such as melatonin and N-salicyloyl tryptamine analogs also exhibiting multi-functional neurological activities. The MTDL generation process is a knowledge-based approach, as it involves modifying a scaffold with known biological activities. The MTDL compounds were synthesized by reacting tryptamine nuclei with various phenacyl, naphthyl, and benzoyl halides (chlorine or bromine). A non-cleavable linked pharmacophore was formed by incorporating three pharmacophores: the parent scaffold, the tryptamine, the linker, and the lipophilic aromatic ring.

Three of the twelve compounds, **SR25**, **SR42**, and **SR10**, exhibited significant and superior AChE inhibitory activity compared to the standard donepezil. **SR25** derivative activity is almost 11-fold higher than donepezil, with an IC50 of 0.17 ± 0.02 µM, while donepezil has an IC50 = 1.96 ± 0.41 µM. The meta-dinitrobenzoyl derivative **SR42** illustrated the second-best activity with an IC50 of 0.70 ± 0.21 µM. The SR10 with para-fluoro phenacyl derivative showed activity with an IC50 of 1.0 ± 0.080 µM.

All the tryptamine derivatives in this work exhibited superior activity for the MAO-B inhibition than the parent compound, tryptamine. Compound **SR42** illustrated higher inhibitory effects against MAO-B with an IC50 = 43.21 ± 0.46 µM. Also, compound **SR25** showed good MAO-B with an IC50 = 85.1 ± 0.26 µM. The placement of the nitro group affected the level of MAO-B inhibition, with the meta position in compound **SR22** exhibiting superior activity compared to the para (**SR23**) or ortho (**SR21**) positions.

Assessment of in vitro human recombinant COX-2 inhibition (COX-2) revealed that the derivatives exhibited inhibition levels between 34.43% and 84.08% at a concentration of 100 µM. The celecoxib (87.86 ± 0.63%) and indomethacin (87.03 ± 0.57%) standards exhibited more pronounced inhibition compared to the derivatives (Table 7).

**Figure 3:**
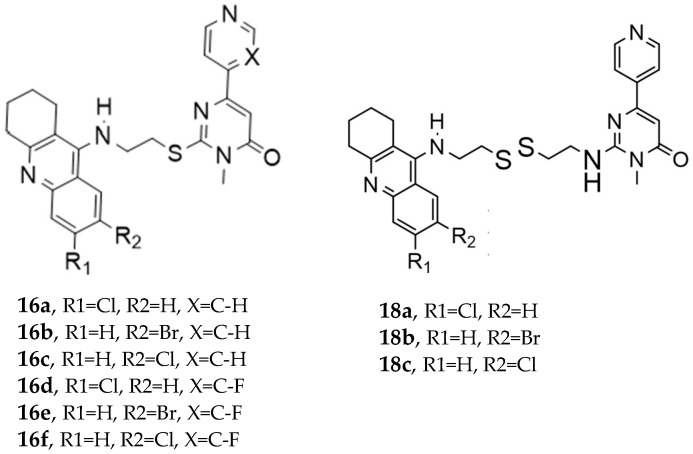
Novel tetrahydroacridin hybrids.

In this paper [61], Wu et al. designed a set of novel tetrahydroacridin hybrid compounds through a knowledge-based approach by combining the well-known AChE inhibitor tacrine with a GSK-3β inhibitor pyrimidone scaffold (Figure 3). The design utilized a non-cleavable linked pharmacophore, connecting the two scaffolds via sulfur-containing linkers—cysteamine or cystamine groups—to create dual AChE/GSK-3β inhibitors.

Compounds (**18a**–**18c**) incorporating the cysteamine group showed greater AChE activity than compounds with a cysteamine linker (series **16a**–**16f**). Specifically, compound **18a** (R1 = Cl, R2 = H, cystamine linker) exhibited the most potent AChE inhibitory activity, with an IC50 value of 0.047 ± 0.002 µM, outperforming tacrine.

Regarding GSK-3β inhibition, as determined by an isolated human recombinant enzyme (GSK-3β), the hybridization with tacrine enhanced the compound’s affinity for GSK-3β. Substituting the alkyl linker with cysteamine or cystamine groups preserved the inhibitory activity of GSK-3β, and introducing a fluorine atom into the pyridine ring significantly enhanced GSK-3β inhibitory activities. The most potent derivatives against GSK-3β were both cystamine linkers **18b** (R1=H, R2=Br, cystamine linker) and **18c** (R1=H, R2=Cl), with IC50 values of 0.37 ± 0.02 µM10 and 0.42 ± 0.03 µM10, respectively (Table 8).

This study by Nagani et al. [62] focuses on the design of novel MTDLs, assessing several inhibitory factors effective against AD (Table 9). The team employed a knowledge-based approach, designing compounds that incorporated piperazine and quinoline scaffolds via a linker in a non-cleavable linked pharmacophore mode. The design aimed to integrate AChE/BuChE inhibition, metal chelation, and antioxidant properties into a single molecule. Substitution of an electron-releasing group (OCH3) or a small atom (F) at the fourth position of the benzyl ring typically led to enhanced activity in the structure–activity relationship (SAR) against AChE, compared to unsubstituted derivatives. The presence of electron-withdrawing groups at the fourth position in the series, specifically chlorine or fluorine atoms, significantly enhanced activity when combined with a 4-methoxybenzyl ring.

The highest AChE inhibitory activity (hAChE) was observed with compound **95** containing a 4-chloro-aniline moiety and a 4-methoxybenzyl group, with an IC50 value of 3.013 µM. Moreover, compounds (**81**, **82**, and **78**) also illustrated AChE inhibition, offering 50% inhibition at concentrations of 8.06, 21.85, and 30.92 µM, respectively. The novel compounds in this study exhibited significant in vitro (eqBuChE) activity against BuChE, particularly those substituted with an o-methoxy group on the aniline ring. The highest BuChE inhibition was observed with compound **83**, an analogue featuring 2-methoxyaniline and 4-fluorobenzyl substituents, with an IC50 value of 1.888 µM. Additionally, compounds **92** and **74** exhibited strong BuChE inhibition, with IC50 values of 2.217 µM and 3.732 µM, respectively.

Regarding metal chelation, the synthesized compounds tended to bind with metals. The compounds also demonstrated metal chelation capabilities, attributed to the 8-hydroxyquinoline moiety, binding various metal ions including Fe^+2^, Fe^+3^, Zn^+2^, Cu^+2^, and Al^+^. All synthesized compounds in this study displayed significant antioxidant properties. At a concentration of 100 µM, compound **78** displayed 55.17% inhibition with an IC50 value of 73.12 µM. Similarly, compound **83** showed 52.91% inhibition with an IC50 of 81.65 µM, and compound **86** showed 51.4% inhibition with an IC50 value of 90.73 µM (Table 9).

**Figure 4:**
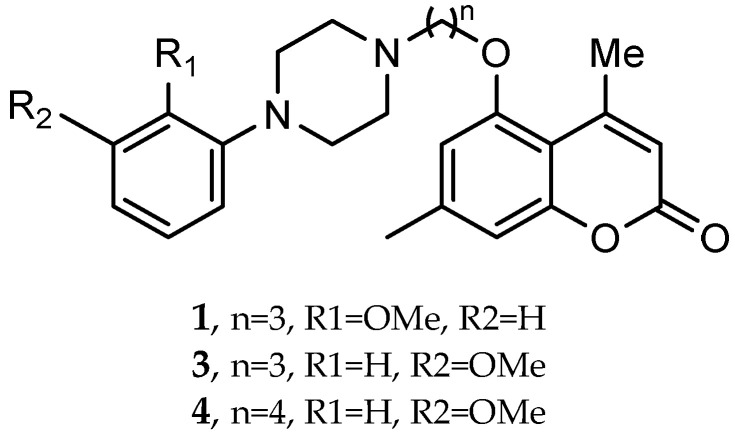
Novel coumarin-O-phenylpiperazine hybrids.

A series of novel compounds that combine a coumarin scaffold with an O-phenylpiperazine moiety via varying linker lengths was studied by Żołek et al. [63]. The team designed and evaluated twelve novel compounds for their potential inhibitory effects against hAChE, human monoamine oxidase A (hMAO-A), and human monoamine oxidase B (hMAO-B) enzymes (Figure 4). A knowledge-based approach counts on standing knowledge of the targets and known active compounds. Additionally, it employs a non-cleavable linked-pharmacophore mode, which connects two distinct pharmacophores: the coumarin scaffold and the O-phenylpiperazine moiety, joined by stable three- or four-carbon alkyl chains.

Compounds **10**, **11**, and **12** 8-acetyl derivatives showed the highest activity, with IC50 values in the low micromolar range. Compound **10** (8-acetyl-7-{4-[4-(2-methoxyphenyl)piperazin-1-yl]butoxy}-4-methylchromen-2-one) was the most potent AChE inhibitor, with an IC50 value of 1.52 ± 0.66 µM. Compounds **11** and **12** had IC50 values of 2.80 ± 0.69 µM and IC50 4.95 ± 0.48 µM, respectively. Activity against hMAO-A, as determined by an isolated human recombinant enzyme, showed that compounds **11** and **12** displayed effective inhibitory properties with IC50 values of 6.97 ± 0.76 µM and 7.65 ± 0.36 µM, respectively. Activity required a compound consisting of a (3-methoxyphenyl)piperazine linked by a three- or four-carbon chain. On the other hand, 2-methoxyphenyl piperazine moiety analogs (compounds **9** and **10**) had poor MAO-A activity. Activity against hMAO-B showed that compounds **1**, **3**, and **4** exhibited the highest activity, demonstrating significant potency in inhibition. The most active compound against hMAO-B was compound **3** with an IC50 value of 1.88 ± 0.45 µM (Table 10).

**Figure 5:**
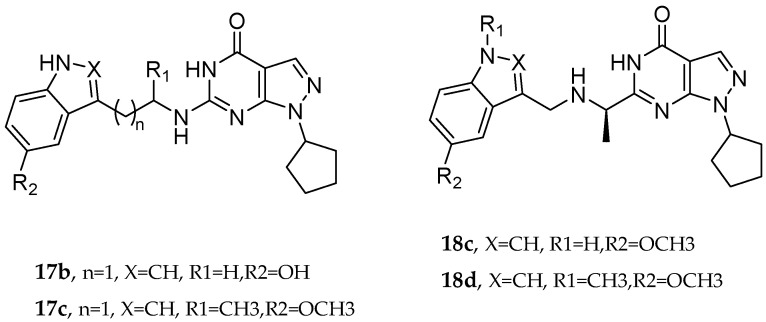
Pyrazolopyrimidinone derivatives.

Q. Zhou et al. and their coworkers [64] designed, synthesized, and evaluated a set of novel pyrazolopyrimidinone derivatives as MTDLs for AD, as shown in Figure 5. Based on a knowledge-based approach, they aimed to combine the potential of enhancing neuronal signaling by inhibiting Phosphodiesterase-9 (PDE9) with the antioxidant properties of melatonin in these novel compounds. The obtained novel MTDL compounds were synthesized by covalently linking the pyrazolopyrimidinone scaffold with the melatonin fragment via a linker.

Most of the novel compounds in this study exhibited moderate to high inhibition against PDE9 using recombinant enzyme (in vitro). Compound **17c** illustrated significant Phosphodiesterase 9 (PDE9) inhibitory activity with an IC50 value of 1.8 nM. Other compounds, such as **17b** and **17c**, also demonstrated notable inhibitory activity against PDE9, with IC50 = 91 nM and IC50 = 89 nM, respectively. The antioxidant properties of these compounds were assessed via the oxygen radical absorbance capacity (ORAC)-FL test. Melatonin’s ORAC value is reported as 1.85 ± 0.04 Trolox equivalents. Compounds **17d**, **18c**, and **18d**, substituted with a methoxy group on the indole ring, exhibited notable ORAC values, with compound **17d** displaying a 2.60-fold, compound **18c** a 1.61-fold, and compound **18d** a 1.09-fold increase in Trolox equivalents. Additionally, compound **17b** with a hydroxyl-indole moiety exhibited good antioxidant activity with an ORAC value of 2.00-fold that of Trolox equivalents (Table 11).

**Figure 6:**
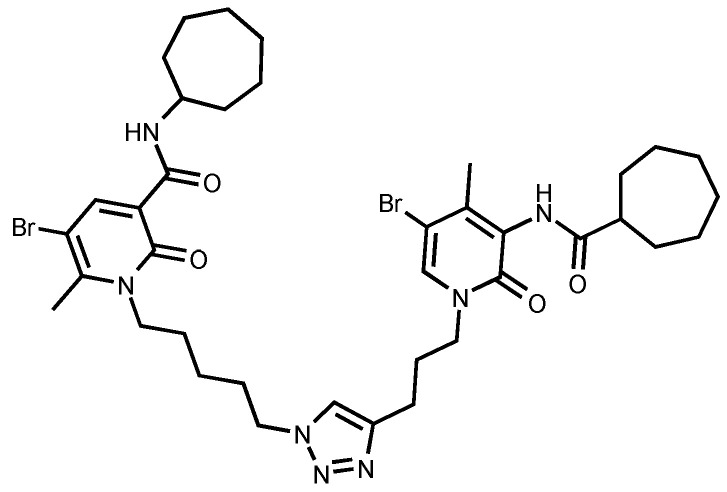
FD22a structure.

The research by Polini et al. [65] explored the capability of the novel cannabinoid receptor type II (CB2R) bitopic/dualsteric ligand (**FD22a**) as a multitarget medication for NDDs (Figure 6). Researchers targeted cannabinoid receptor type II (CB2R), which plays a role in neuroinflammatory processes and microglial cell activation. **FD22a** is a type of bitopic ligand, designed to bind to two different sites on the same receptor.

**FD22a** pretreatment effectively inhibited Aβ25–35-induced toxicity. In both HMC3 and U87-MG cell lines, **FD22a** at 1 µM significantly increased cell survival rates compared to cells exposed only to the toxic peptide.

The activation of microglial cells and the subsequent release of inflammatory markers, including TNF-α and IL-6, are crucial elements of neuroinflammation associated with AD. **FD22a** effectively mitigated the rise in these proinflammatory cytokines triggered by Aβ25–35 in HMC3 and U87-MG cell lines. Administering **FD22a** alone did not lead to a notable increase in cytokine production. The protective effect of **FD22a** against inflammation in HMC3 cells triggered by Aβ was suppressed mainly by SR144528, a CB2R antagonist, suggesting that this receptor mediates **FD22a**’s anti-inflammatory actions. Pretreatment with **FD22a** before the application of Aβ25–35 led to a notable increase in IL-10 production by HMC3 and U87-MG cells, in addition to decreasing the levels of proinflammatory cytokines. Impaired function in the autophagy–lysosomal pathway (ALP) is a common characteristic among various neurodegenerative disorders, such as AD.

The presence of the β25–35 peptide in U87-MG cells resulted in a substantial reduction in the levels of pro-autophagy genes (LC3, SIRT1, and SIRT6). At the same time, the expression of negative regulators of autophagy (mTOR, SIGMAR1, and SIRT5) was markedly increased. The application of **FD22a** at a concentration of 1 µM effectively inhibited the autophagy suppression induced by β-amyloid contact. However, **FD22a** alone did not result in substantial transcriptional effects related to autophagy. The analysis of quantitative proteomics on U87-MG cells, which had been treated with **FD22a** and Aβ25–35, in contrast to just Aβ25–35, demonstrated that **FD22a** significantly activates the ALP pathway by promoting the dephosphorylation and activation of the master regulator TFEB, likely through the reduction in active p-mTOR.

### 5.2. Fused Pharmacophore Strategies

The study of Bajad et al. [66] aimed to design and synthesize novel MTDLs by structurally optimizing the chalcone scaffold into a novel series of pyrazoline derivatives bearing an N-aryl piperazine moiety (Table 12). Earlier studies showed that chalcone derivatives have inhibitory potential against key AD targets such as AChE, BuChE, BACE1, and Aβ aggregation [67]. This study synthesized and evaluated a series of twenty-six novel pyrazoline analogs (compounds **32**–**57**) for their AD treatment potential. Compound **48** appeared as the most promising multi-target directed ligand. The pyrazoline core and the N-aryl piperazine are directly integrated within a single molecular framework. This aligns with the fused-pharmacophore mode, where MTDLs have partially overlapped pharmacophores.

Evaluation of cholinesterase inhibition revealed that N-benzyl piperazine derivatives generally showed more potent activity than phenyl piperazine derivatives. Compounds **48**–**57**, featuring the N-acetylated pyrazoline scaffold, particularly in conjunction with benzylpiperazine, significantly enhanced their ChE inhibitory potency. Compound **48** exhibited potent dual inhibitory activities with IC50 values of 2.89 ± 0.706 μM for AChE and 0.151 ± 0.089 μM for BuChE. Also, compound **56** showed potent inhibitory activity on AChE with IC50 = 2.831 ± 0.528 μM, and on BuChE with IC50 = 0.569 ± 0.031 μM. Compounds with dual potent AChE and BuChE inhibitory profiles (48, 53, 54, 56) were chosen for studying their potential to inhibit hBACE-1. Compound **48** demonstrated significant inhibition of hBACE-1, with a value of 36.64 ± 1.343% at 10μM, and a potent anti-aggregation profile against Aβ1–42 in both self-induced aggregation using the Thioflavin T assay and AChE-induced aggregation. At a concentration of 20 μM, compound **48** exhibited maximal inhibitory activity in both self-induced and AChE-induced aggregation experiments, indicating its promising multi-target activity (Table 12).

In [68], V. Kumar et al. designed, synthesized, and evaluated novel 4-N-substituted-2-derivatives as MTDLs for treating AD (Table 13). The novel MTDLs were designed for dual inhibition of AChE and MAO-B enzymes, as well as the inhibition of Aβ42 aggregation and the generation of ROS. The work combined a rigid 2-phenylquinazoline nucleus with various pharmacophores, including a tertiary nitrogen and a propargyl ether pharmacophore. This was based on established knowledge of the antioxidant activity of the quinazoline moiety, as well as previous studies that demonstrated the crucial role of propargyl groups and tertiary nitrogen moieties in AChE and MAO inhibition [60,69]. The novel compounds **VAV-8** and **VAV-19** were recognized as the most potent MTDLs out of 20 newly synthesized quinazoline derivatives. Structures VAV-8 and VAV-19, with a morpholine or benzylpiperazine at C4 and an O-propargyl-phenyl at C2 of the quinazoline, illustrate that the key elements, tertiary nitrogen and propargyl ether, are directly incorporated into the central quinazoline scaffold, rather than being separate entities joined by a non-pharmacophoric linker. The design strategy aligns with the fused-pharmacophore mode.

All synthesized compounds showed selective AChE inhibition over BuChE, mainly in the micromolar range. VAV-8 was the most potent and selective AChE inhibitor with an IC50 value of 0.17 ± 0.07 µM. VAV-19 also showed selective AChE inhibition with an IC50 value of 0.177 ± 0.05 µM. Compound **VAV-8** exhibited potent MAO-B inhibition with an IC50 value of 0.078 ± 0.009 µM. **VAV-19** presented MAO-B inhibition with an IC50 value of 6.06 ± 0.69 µM. Most compounds were selective MAO-B inhibitors, with VAV-4 showing some MAO-A inhibition at 0.898 ± 0.15 µM.

The novel MTDLs were evaluated for their ability to inhibit Aβ42 aggregation at a concentration of 10 µM. **VAV-8** and **VAV-19** exhibited moderate anti-aggregation potential by inhibiting the Aβ42 aggregation with 23.54% and 33.01% inhibition, respectively, after 72 h. The standards resveratrol and curcumin showed higher inhibition (58.01% and 49.61%, respectively). Regarding ROS inhibition, compounds **VAV-8** and **VAV-19** significantly reduced intercellular ROS levels in human neuroblastoma (SH-SY5Y) cells. At 1 µM concentration, **VAV-8** showed a 48.0% decrease in ROS, whereas VAV-19 exhibited a 57.4% reduction. At concentrations of 5 µM and 25 µM, **VAV-8** decreased ROS levels to 56.6% and 40.7%, respectively (Table 13).

**Figure 7:**
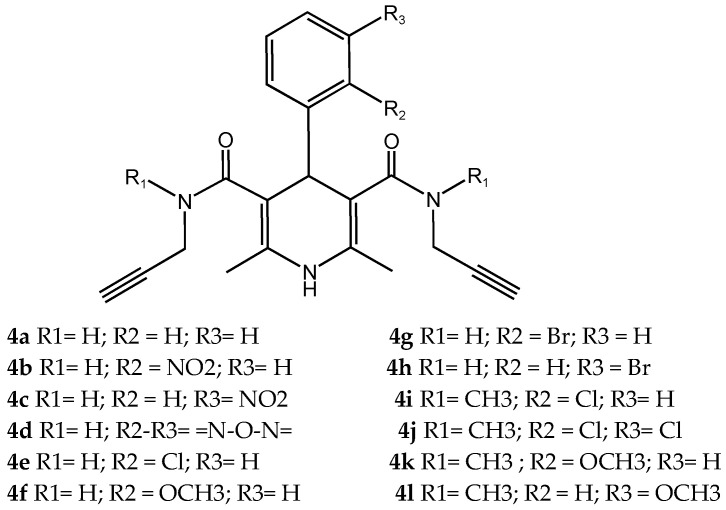
Novel MTDLs **4a**–**4l**.

Pachón-Angona et al. [70] describe the design, synthesis, and biological evaluation of a series of 12 novel MTDLs for AD (Figure 7). The compounds (**4a**–**l**) were synthesized via a modified one-pot three-component Hantzsch reaction, where dihydropyridines (DHPs), which exhibit inherent calcium channel modulation activity, were combined with propargyl amide residues. This reaction directly incorporates propargyl amide residues as electrophilic substructures into the dihydropyridine (DHP) skeleton, aligning with the fused-pharmacophore mode.

Novel compounds in this study exhibited antioxidant activity using the ORAC assay, with results quantified in Trolox equivalents (TE), except for compound **4k**. Compound **4i** performed significantly with 1.93 TE, then **4j** with 1.82 TE, and **4c** with 1.54 TE. The results indicate that the novel compounds were less active by a factor of 1.3 times than the positive control, melatonin. Electron-withdrawing groups, such as halogen or nitro, substituted on the aromatic ring appeared to improve the antioxidant activity.

The Nrf2 Transcriptional activation was evaluated using a cell-based luciferase assay in AREc32 cells, with the positive control of melatonin yielding a CD value of 66.4. The novel compounds showed no cytotoxicity up to 100 µM, and up to 150 µM for compounds **4b**, **4i**–**l**. Compounds **4b** and **4i**–**l** significantly induced the Nrf2 pathway at concentrations up to 150 µM. The CD values of the compounds were 96.0 µM for **4i**, 82.8 µM for **4b**, 81.7 µM for **4l**, 77.2 µM for **4j**, and 69.4 µM for **4k**, while compounds **4a**, **4c**–**h** did not show significant Nrf2 activation. Compared to melatonin, compound **4k** displayed similar activity levels, whereas compounds **4b**, **4i**, **4j**, and **4l** were significantly less active, with activity reduced by 1.2 to 1.5 times. Generally, compounds with a tertiary amide group had the capacity to induce the Nrf2 pathway, and the most active analogue was substituted with an ortho-chloro group **4i**. However, primary amide derivatives did not show any activity, except for compound **4b**, which contained an ortho-nitro group substitution (Table 14).

### 5.3. Merged Pharmacophore Strategies

Pinheiro et al. [71] developed first-in-class multitarget compounds that act as dual agonists of the G protein-coupled receptor 40 (GPR40) and Histone Deacetylase 6 (HDAC6) inhibitors. Researchers applied the merging approach to design a novel series of hydroxamic acid derivatives (compounds **4a–i**). The design was guided by the bioisosteric relationship between the carboxylic acid group, commonly found in GPR40 agonists, and the hydroxamic acid, a zinc-binding group widely used in HDAC inhibitors. A conformational restriction was carried out on compound **5a** by constructing a double bond between the hydroxamic acid group and the phenyl ring to assess its impact on activity. Also, the structural pattern of compounds **6a** and **6g** changed from 1,4- to 1,3-substitution with conformational restriction to induce allosteric and agonist activity toward GPR40. The novel compounds are designed using a merged pharmacophore mode, where the acidic head (hydroxamic acid in place of carboxylic acid) and the lipophilic cap group of the GPR40 reference agonist (GW9508) are directly linked to the known attributes of HDAC inhibitors.

All compounds showed activity against GPR40, with Emax values spanning from 55% to 117% compared to the reference agonist GW9508 (100%), except for compounds **6g** and **4b**. Compound **4a**, with the bioisosteric replacement of carboxylic acid with a hydroxamic acid, had an EC50 of 9.5 nM compared to GW9508 (EC50 = 7 nM). Compound **4b**, featuring a bulky diphenyl cap group, exhibited low activity due to steric hindrance. Compound **5a**, with conformational restriction, retained high GPR40 activity and was the most potent GPR40 agonist within its series (EC50 = 5.4 nM).

Regarding HDAC6 inhibition, only compounds with greater than 50% inhibition were further tested to determine their IC50 values. Compound **4e** was the most potent inhibitor of HDAC6 with an IC50 value of 73 nM. The conformational restriction observed in compound **5a**, with an IC50 value of 267 nM between the phenyl ring and the hydroxamic acid, led to positive inhibition of HDAC6. However, the most potent derivatives were found with an unrestricted conformation of the series (**4a**–**i**), such as compound **4d** with an IC50 value of 160 nM. Derivatives with a single phenyl group (**4a**–**i**) were more potent, with an IC50 value of 157 nM (**4i**), compared to those with two phenyl groups (**4h**, IC50 = 551 nM), indicating that steric hindrance caused by the bulky groups played a role. The 1,4-substitution pattern compounds **4a**/**5a** exhibited better HDAC6-suppressing activity than the 1,3 pattern, possibly due to hydrogen bonding with Ser531, which is a critical factor for efficacy and specificity.

In vitro cell-based assays using SH-SY5Y neuroblastoma cells showed that compounds **4c**, **4d**, and **4e** increased histone H3 acetylation. Compounds **4d** and **4e** also caused a notable rise in α-tubulin acetylation (a biochemical marker for HDAC6 activity) at 1 μM. Activation of GPR40 in human neuroblastoma cells led to ERK phosphorylation. It was also observed in SH-SY5Y cells when treated with compounds **4d** and **4e**, resulting in a greater increase in ERK phosphorylation compared to untreated cells. Compound **4e** showed a slight increase, even at a concentration of 0.1 μM (Table 15).

Said et al. and their co-workers [72] designed, synthesized, and evaluated a novel series of 3-hydrazinyl indole phenacetamide derivatives to address AD and neuroinflammation by targeting AChE, BChE, and BACE1 enzymes (Table 16). Their design used a merged pharmacophore approach, integrating core structural features from donepezil and the BACE1 inhibitor LY2886721 into a single bicyclic indole scaffold. The bicyclic indole moiety was chosen as the core structural component due to its electronic and steric similarity to the indanone ring in donepezil and the furothiazine ring in LY28867215. The indole was used as a spacer and was placed at positions 1 and 3 with acetamide and hydrazinyl moieties. In addition, the terminal aromatic rings were substituted with electronic and lipophilic groups to optimize activity.

The researchers synthesized a series of fifteen novel 3-hydrazinyl indole phenacetamide derivatives (**5a**–**o**). Compound **5a**, the N-phenylacetamide indole derivative bearing an unsubstituted phenylhydrazinyl side chain, was identified as the most promising novel MTDL. In vivo studies against carrageenan-induced rat paw edema showed that all compounds (**5a**–**o**) exhibited significant anti-inflammatory activity. When compared to indomethacin with 72.26% inhibition, numerous derivatives presented superior activity, reaching up to 91.61% inhibition for compound **5f**. Other compounds such as **5a**–**c**, **5j**, and **5o** also showed inhibition activity with 76.59%, 80.45%, 73.90%, 72.98%, and 72.98%, respectively.

The AChE inhibition activity of novel compounds in this study is linked to the presence of an unsubstituted phenyl hydrazinyl side chain paired with an unsubstituted, p-methyl, or p-chloro phenacetamide moiety. Compounds **5a**–**c**, **5j**, and **5o** displayed good inhibitory activity at 500 μM, for example, **5a** showed 68.97% inhibition, **5c** 66.15%, **5j** 63.97%, and **5o** 59%. When compared to donepezil (IC50 = 0.304 μM), many compounds in the in vitro AChE and BChE inhibition assays performed better than donepezil. Compound **5o** exhibited the most potent AChE inhibitory activity with an IC50 value of 0.195 μM. Also, compounds **5a** and **5j** had IC50 values of 0.268 and 0.302 μM, respectively. For BChE inhibitory activity, all compounds showed comparable activity; compound **5o** was the most potent with an IC50 value of 2.321 μM.

SAR studies revealed that an unsubstituted phenyl hydrazinyl side chain was optimal for AChE and BChE inhibition when paired with an unsubstituted phenacetamide group, confirming the effectiveness of the merged design (Table 16).

### 5.4. Natural Product Scaffolds

Vitale et al. [73] highlight natural compounds as a valuable source of bioactive molecules due to their diverse chemical properties and functional groups, which predispose them to interact with numerous related targets of AD (Table 17). The research team examined two naturally occurring compounds from the cannabis sativa plant, cannabidiolic acid (CBDA) and cannabigerolic acid (CBGA), as MTDLs for AD. Building on previous work demonstrating their dual PPARα/γ agonist properties, this study extended the investigation to their effects on AD-related enzymes and pathology [74].

CBDA and CBGA both showed BACE-1 inhibitory effects in a dose-dependent manner; CBGA, with an IC50 value of 1.4 ± 0.1 μM, proved to be roughly four times more potent than CBDA, with an IC50 value of 6.1 ± 0.2 μM, in suppressing cellular mouse BACE-1 activity. Both compounds inhibited the self-aggregation of the Aβ 1–40 peptide (human β-amyloid peptide (1–40)); for CBGA, the IC50 was 47.7 ± 2.1 μM, while for CBDA, the IC50 was 57.5 ± 0.8 μM. Both compounds demonstrate a direct effect on counteracting β-amyloid formation and a BACE-1-mediated effect. The two compounds, CBDA and CBGA, with low micromolar concentrations, were successful in inhibiting the AChE (Electrophorus electricus) and BChE (equine serum) enzymes (Table 17). Computational studies suggested that CBDA and CBGA inhibit AChE competitively and BuChE non-competitively.

In vivo studies showed that repeated treatment with CBDA and CBGA (10 mg/kg, intraperitoneally) in an AD mouse model (CD1 male mice) improved cognitive function deficits caused by β-amyloid peptide in mice. Both CBDA and CBGA improved cognitive function in the Novel Object Recognition test and restored hippocampal long-term potentiation (LTP). CBGA administration had a substantial impact on depressive-like behavior, which was assessed by immobility time in the Tail Suspension Test; in contrast, CBDA did not exhibit a significant effect on this behavior.

The above example demonstrates the recent MTDLs, which were primarily developed using knowledge-based (pharmacophore-based) approaches to combine multiple activities into a single molecule for AD. This strategy involves combining two or more well-known pharmacophores into a single molecular entity to integrate multiple activities. The resulting MTDLs are classified into categories such as linked, fused, and merged pharmacophores based on how these key pharmacophores are integrated. Among the most active MTDL agents highlighted in this review are compounds **6h**, **VAV 8**, **5a3**, and **21**, as shown in Table 18.

## 6. Conclusions

AD represents a global health warning that affects all levels of society due to its profound social and economic burden. With millions currently affected and a projected 68% rise in dementia cases in low- and middle-income countries by 2050, the need for effective treatments is urgent. While current FDA-approved medications, including AChE inhibitors, memantine, and the newer anti-amyloid antibodies, offer benefits, they primarily manage symptoms or target a single pathway, creating a significant therapeutic gap. In response, the MTDL strategy has emerged in recent years. However, this strategy has not yet been fully recognized or implemented by the mainstream. One reason might be that the single-target approach is the standard, conventional method for traditional drug development, which is more acceptable to the FDA approval process. Another reason may lie in the challenges in developing such a strategy and in the complexity of evaluating the mechanisms of drug actions. Despite these ongoing challenges and the complexity of regulatory approval, MTDLs are well-positioned to become the key foundation for future AD treatments. Their innovative, multi-targeted approach is crucial to overcoming the limitations of current symptomatic treatments and effectively addressing the disease’s complex, multifactorial nature. The primary goals of MTDLs’ methods are to provide enhanced therapeutic benefits, improve effectiveness, increase safety, reduce drug resistance, and yield more reliable pharmacokinetic and pharmacodynamic outcomes. The majority of the MTDL’s development process discussed in this review is based on molecular hybridization. By combining two or more known pharmacophores into a single molecular entity, primarily through non-cleavable linked pharmacophores, fused methods, and merged pharmacophores, multi-functional activity can be achieved. The multi-targeted approach provides broad therapeutic potential against various AD hallmarks, such as cholinesterase inhibition, Aβ aggregation, MAO inhibition, and oxidative stress.

## Figures and Tables

**Table 1:** Nrf2 induction potencies of compounds **4a–4h** and the reference compound tBHQ, determined using in vitro Nrf2/ARE-luciferase reporter cells [54].

Novel MTDLs	Nrf2 Induction Potencies CD (µM)
**4a**	1.0 ± 0.1
**4b**	0.4 ± 0.1
**4c**	0.3 ± 0.1
**4d**	0.7 ± 0.1
**4e**	1.0 ± 0.1
**4f**	0.4 ± 0.1
**4g**	0.7 ± 0.2
**4h**	0.5 ± 0.1
tBHQ	0.6 ± 0.1

**Table 2:** AChE and BuChE inhibitory activities, as well as OH scavenging abilities, were studied using in vitro enzymatic and chemical assays of compounds **1–2** [55].

Novel MTDLs	(AChE) Inhibition (IC50, μM)	(BuChE) Inhibition (IC50, μM)	OH Scavenging Abilities (IC50, μM)
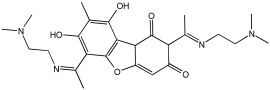 **1**	0.713 ± 0.005	5.934 ± 0.141	0.865 ± 0.025
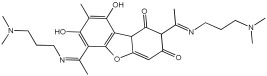 **2**	0.143 ± 0.006	0.356 ± 0.008	0.572 ± 0.004
Tacrine	0.0145 ± 0.001	0.003 ± 0.000	
Trolox			0.63 ± 0.010

**Table 3:** IC50 values of the novel compounds against hAChE, hBuChE, GSK-3β, Aβ aggregation inhibition, and tau protein aggregation inhibition determined using in vitro enzymatic and aggregation assays, in comparison with donepezil and rivastigmine as reference standards [56].

Novel MTDLs	hAChE Inhibition (IC50 nM ± SD)	hBuChE Inhibition (IC50 nM ± SD)	GSK-3β Inhibition (IC50 nM ± SD)	(Aβ) Aggregation Inhibition IC50 uM	Tau Protein Aggregation Inhibition IC50 uM
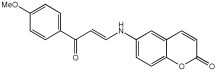 **6c**	28.88 ± 3.19	131.90 ± 8.17	51.42 ± 2.94	22.45 ± 1.05	31.22 ± 1.90
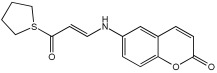 **6h**	26.03 ± 3.99	90.02 ± 6.71	26.91 ± 2.19	35.04 ± 1.64	56.31 ± 3.43
Donepezil	31.54 ± 2.16	614.50 ± 7.30	219.10 ± 5.82	75.31 ± 3.53	120.1 ± 7.32
Rivastigmine		107.40 ± 7.49	−		

**Table 4:** MAO-A, MAO-B, and AChE inhibition activities of novel benzylpiperidine–isatin hybrids studied using in vitro enzymatic assay [57].

Novel MTDLs	MAO-A IC 50(μM) ± SEM ^a^	MAO-B IC 50(μM) ± SEM ^a^	(AChE) Inhibition IC 50(μM) ± SEM ^a^
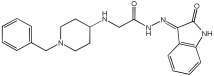 **4**	0.22 ± 0.02	0.057 ± 0.001	0.27 ± 0.01
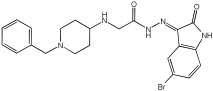 **5**	0.65 ± 0.05	39.81 ± 1.04	3.25 ± 0.14
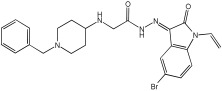 **11**	9.67 ± 0.33	0.61 ± 0.03	2.16 ± 0.08
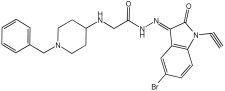 **12**	0.12 ± 0.07	5.73 ± 0.06	2.32 ± 0.17
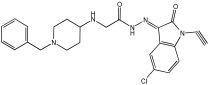 **14**	0.108 ± 0.004	0.60 ± 0.003	0.034 ± 0.002
Donepezil			0.084 ± 0.002

^a^ Each IC50 value is presented as the mean± SEM (*n* = 2 or 3) and indicates the assay concentration of the test compound that results in 50% inhibition of enzyme activity [57].

**Table 5:** In vitro eeAChE, eqBChE, and MAOs inhibitory activities of cinnamic acid natural product-based MTDLs [58].

Novel MTDLs	AChE IC50 (μM ± SD)	BChE IC50 (μM ± SD)	MAO-A IC50 (μM ± SD)	MAO-B IC50 (μM ± SD)
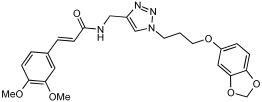 **16**	4.59 ± 0.87	13.24 ± 0.56	30.78 ± 0.94	32.02 ± 0.59
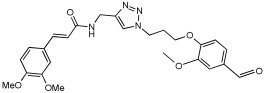 **14**	7.51 ± 0.5	21.6 ± 0.85	61.44 ± 0.44	63.44 ± 0.39
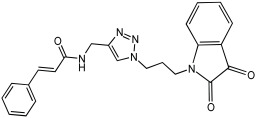 **18**	6.42 ± 0.16	35.3 ± 0.38	61.2 ± 0.42	46.59 ± 0.72
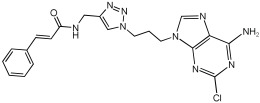 **21**	0.37 ± 0.24	13.53 ± 0.52	33.68 ± 0.57	64.09 ± 1.39
	Donepezil 6.01 ± 0.2	Rivastigmine 5.88 ± 1.86	Methylene blue 143.6 ± 22.1	Biperiden HCl 66.4 ± 1.47

**Table 6:** Calculated inhibitory capacity of the RIV-IND hybrids (and reference compounds) for Aβ42 aggregation and MAO, determined using in vitro aggregation and enzymatic assays [59].

Novel MTDLs	Aβ42 Self-Aggr. Inhib. (%)	Aβ42 Cu-Ind Aggr. Inhib. (%)	hMAO-A IC50 (μM) or % Inhibition at 10 μM	hMAO-B IC50 (μM) or % Inhibition at 10 μM
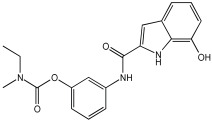 **5a3**	25.0	48.3	6.66 ± 0.74	3.85 ± 0.10
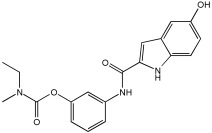 **5a2**	50.3	73.5	28%	36%
Curcumin	61.3	53.0	-	-
(R)-(-)-Deprenyl	__	__	18 ± 1	0.049 ± 0.009
Clorgyline	__	__	0.00200 ± 0.00015	2.44 ± 0.49

**Table 7:** In vitro AChE inhibition, MAO-B inhibition, and COX-2 inhibition activities studied using in vitro enzymatic assays [60].

Novel MTDLs	AChE	(MAO-B)	(COX-2) %Inhibition ± SD
	(IC_50_ ± SD (µM))		
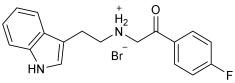 **SR10**	1.00 ± 0.08	216.10 ± 0.29	72.90 ± 2.42
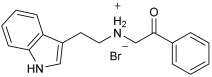 **SR25**	0.17 ± 0.02	85.10 ± 0.26	72.43 ± 2.36
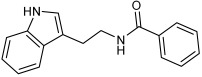 **SR42**	0.70 ± 0.21	43.21 ± 0.46	75.16 ± 2.30

**Table 8:** Inhibitory activities of tetrahydroacridin hybrids against AChE and GSK-3β studied using in vitro enzymatic assays [61].

Novel MTDLs	(AChE) Inhibition (IC50, μM) ± SEM	GSK-3β (IC50, μM) ± SEM
**16a**	2.30 *±* 0.14	15.10 *±* 0.90
**16b**	20.00 ± 1.50	38.50 ± 0.70
**16c**	10.30 ± 0.70	2.28 ± 0.07
**16d**	1.89 ± 0.21	1.69 ± 0.09
**16e**	15.30 ± 0.90	0.94 ± 0.02
**16f**	16.50 ± 0.80	2.44 ± 0.06
**18a**	0.047 ± 0.002	0.93 ± 0.08
**18b**	2.13 ± 0.11	0.37 ± 0.02
**18c**	2.24 ± 0.13	0.42 ± 0.03
Tacrine	0.229 ± 0.01	-

**Table 9:** Anti-AChE, anti-BuChE, and antioxidant potentials of the piperazine–quinoline hybrids, determined using in vitro enzymatic assays [62].

Novel MTDLs	hAChE (IC50 ± SD, μM)	eqhBuChE (IC50 ± SD, μM)	DPPH Antioxidant Potential (IC50, μM)
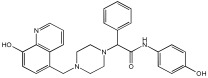 **78**	30.92 ± 0.996	12.42 ± 0.219	156.06
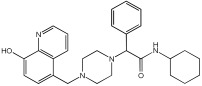 **79**	214.46 ± 0.657	2.28 ± 0.355	237.21
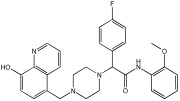 **83**	14.09 ± 0.266	1.888 ± 0.300	162.16
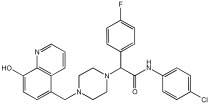 **86**	64.32 ± 0.678	2.02 ± 0.270	179.69
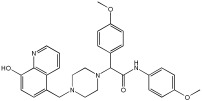 **93**	76.43 ± 0.145	4.953 ± 0.295	-
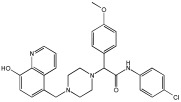 **95**	3.013 ± 0.433	3.144 ± 0.189	211.99
Donepezil	0.054 ± 0.002	-	-
Tacrine	-	0.0047 ± 0.0002	-
Ascorbic acid	-	-	79.37

**Table 10:** Inhibitory activity against hAChE, hMAO-A, and hMAO-B of coumarin-O-phenylpiperazine hybrids, studied using in vitro enzymatic assays [63].

Novel MTDLs	(AChE) % Inhibition at 10 µM ± SD	hMAO-A % Inhibition at 10 µM ± SD	hMAO-B % Inhibition at 10 µM ± SD
**1**	61 ± 1%	44 ± 4%	2.18 ± 0.48
**3**	53 ± 2%	39 ± 5%	1.88 ± 0.45
**4**	56 ± 1%	38 ± 4%	3.18 ± 0.63
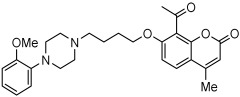 **10**	1.52 ± 0.66	52 ± 4%	24 ± 1%
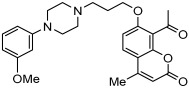 **11**	2.80 ± 0.69	6.97 ± 0.76	32 ± 1%
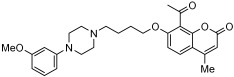 **12**	4.95 ± 0.48	7.65 ± 0.36	57 ± 4%

**Table 11:** Inhibitory activities against PDE9, studied using a recombinant enzyme assay, and ORAC [64].

Novel MTDLs	PDE9 (IC_50_*,* nM)	ORAC µmol of Trolox Equiv/µmol
**17b**	91 ± 4	2 ± 0.27
**17c**	1.8	0.32 ± 0.06
**18c**	194 ± 26	1.61 ± 0.11
**18d**	214 ± 20	1.09 ± 0.02
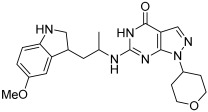 **17d**	89 ± 4	2.6 ± 0.5

**Table 12:** Cholinesterase (eeAChE and eqBuChE) inhibitory profile and BACE-1 inhibitory activity of pyrazoline derivatives, determined using in vitro enzymatic assays [66].

Novel MTDLs	eeAChE IC50 (μM) or % Inhibition	eqBuChE IC50 (μM) or % Inhibition	BACE-1 % Inhibition
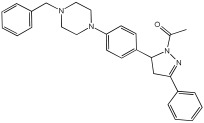 **48**	2.89 ± 0.706	0.151 ± 0.089	36.64 ± 1.343
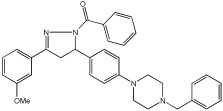 **53**	4.086 ± 0.415	0.247 ± 0.072	26.19 ± 1.635
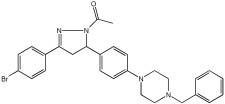 **54**	3.393 ± 0.916	1.079 ± 0.300	41.02 ± 1.290
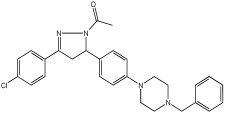 **56**	2.831 ± 0.528	0.569 ± 0.031	34.64 ± 1.198

**Table 13:** Inhibitory potential of the synthesized compounds against eeAChE, eqBuChE, hMAO-A, and hMAO-B enzymes, determined using in vitro enzymatic assays [68].

Novel MTDLs	eeAChE %Age Inhibition at 10 µM	MAO-B %Age Inhibition at 10 µM	MAO-A %Age Inhibition at 10 µM	eqBuChE %Age Inhibition at 20 µM
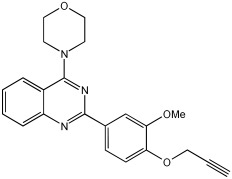 **VAV 8**	96.34% (0.171 ± 0.07)	98.54% (0.078 ± 0.009)	43.11%	7.28%
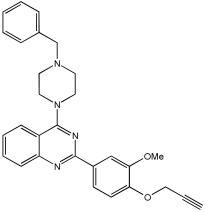 **VAV 19**	95.52% (0.177 ± 0.05)	71.92% (6.06 ± 0.69)	47.01%	7.58%

**Table 14:** Biological activities of compounds **4a**–**l**: in vitro calcium channel blockade using human neuroblastoma cell line SH-SY5Y, AREc32 cell line for Nrf2 induction, and chemical assays of ORAC [70].

Novel MTDLs5	Calcium Channel Blockade% at 10 µM	ORAC Trolox Equiv/± SEM	Nrf2 Induction Potencies CD (µM)
**4a**	12	0.86 ± 0.5	n.d.
**4b**	n.i.	1.36± 0.16	82.8 ± 8.4
**4c**	3	1.54 ± 0.15	n.i.
**4d**	10	1.23 ± 0.23	n.i.
**4e**	n.i.	1.15± 0.02	n.i.
**4f**	19	1.34 ± 0.2	n.i.
**4g**	n.i.	1.43 ± 0.03	n.i.
**4h**	7	1.28 ± 0.05	55.3 ± 7.4
**4i**	11	1.93 ± 0.08	69.3 ± 5.2
**4j**	12	1.82 ± 0.05	n.i.
**4k**	14	n.i.	n.i.
**4l**	15	0.96 ± 0.07	n.i.
nimodipine	37	n.d.	n.d.
melatonin	n.d.	2.45 ± 0.09	n.d.

n.d., not determined, indicates that no experiments were performed. n.i., no inhibition, refers to a residual activity ≥ 95% at 50 µM inhibitor concentration.

**Table 15:** Pharmacological activity data of GPR40 activation, determined using CHO–K1 cells in an in vitro assay, and HDAC6 inhibition, studied using an in vitro enzymatic assay [71].

Novel MTDLs	HDAC6 IC50 (nM)	GPR40 EC50 (nM)	Emax GPR40
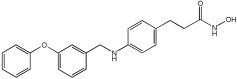 **4a**	18% (1 μM)	9.5	93%
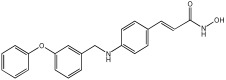 **5a**	267	5.4	88%
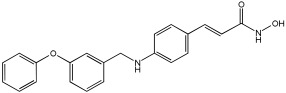 **6a**	1590	683	55%
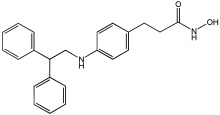 **4b**	31% (1 μM)	ND	−7%
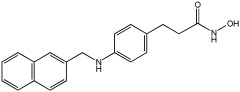 **4c**	301	30.6	109%
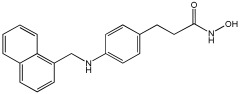 **4d**	160	22.9	113%
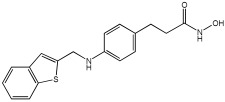 **4e**	73	22.5	95%
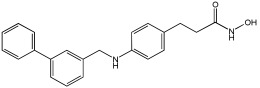 **4h**	551	8.9	109%
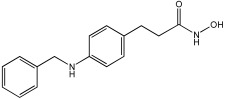 **4i**	157	766	55%
GW9508	–	7.0	100%

**Table 16:** In vivo anti-inflammatory effect of the newly synthesized compounds and indomethacin against carrageenan-induced hind paw edema in rats, and IC50 values of 3-hydrazinyl indole phenacetamide derivatives against AChE and BChE, determined using in vitro enzymatic assays [72].

Novel MTDLs	Edema (mm) ± SEM (% Inhibition) 2 h	(AChE) IC50 ± SD (μM)	(BChE) IC50 ± SD (μM)
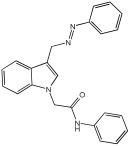 **5a**	76.59%	0.268 ± 0.01	2.777 ± 0.45
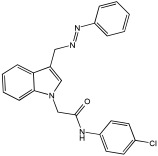 **5b**	80.45%	0.507 ± 0.002	2.925 ± 0.10
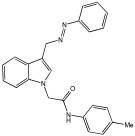 **5c**	73.90%	0.656 ± 0.001	3.131 ± 0.10
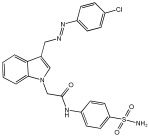 **5j**	74.78%	0.302 ± 0.01	2.908 ± 0.15
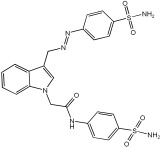 **5o**	72.98%	0.195 ± 0.0001	2.321 ± 0.10
Donepezil		0.304 ± 0.005	1.382 ± 0.01
Indomethacin	72.26%		

**Table 17:** Inhibitory activities of CBDA and CBGA, studied using in vitro enzymatic assays [73].

Novel MTDLs	(AChE) Inhibition (Ki_,_ μM)	(BuChE) Inhibition (Ki_,_ μM)	(BACE1) Inhibition (IC_50,_ μM)	(Aβ) Aggregation Inhibition (%)μM
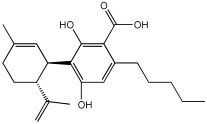 CBD	7.9 ± 4.3	6.7 ± 0.6	6.1 ± 0.2	57.5 ± 0.8
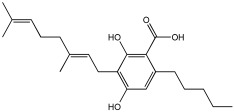 CBGA	10.5 ± 2.9	23.3 ± 12.0	1.4 ± 0.1	47.7 ± 2.1

**Table 18:** Most active MTDLs.

Novel MTDLs	Targets Inhibition
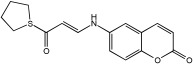 **6h** MTDLs integrate the Coumarin scaffold	hAChE, hBuChE, GSK-3β, (Aβ) aggregation, and tau protein aggregation [56].
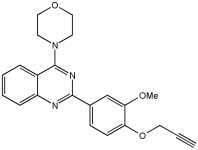 **VAV 8** MTDLs integrate the 2-phenylquinazoline scaffold	eeAChE, MAO-B, MAO-A, and eqBuChE [68]
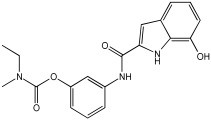 **5a3** MTDLs integrate rivastigmine–melatonin scaffolds	Aβ42, Aβ42, hMAO-A, and hMAO-B [59]
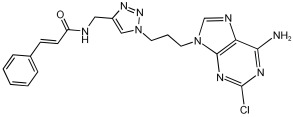 **21** Natural product-based MTDLs integrate cinnamamide scaffold	AChE, BChE, MAO-A, and MAO-B [58]

## Data Availability

No new data were created or analyzed in this study. Data sharing is not applicable to this article.

## Abbreviations

The following abbreviations are used in this manuscript:

MTDLs	Multitarget Directed Ligands
AD	Alzheimer’s disease
AChE	Acetylcholinesterase
BuChE	Butyrylcholinesterase
MAO	Monoamine Oxidase
NDDs	Neurodegenerative diseases
Aβ	Amyloid-Beta
BACE1	β secretase
GSK-3β	Glycogen Synthase Kinase 3β
CDK5	Cyclin-Dependent Kinase 5
APP	Amyloid precursor protein
NFTs	Neurofibrillary tangles
ROS	Reactive oxygen species
pTau	Hyperphosphorylated tau
NMDAR	N-methyl-D-aspartate receptor
Nrf2	Nuclear factor erythroid 2-related factor 2
IC50	Inhibitory concentration 50%
NQO1	NQO1 expression (a downstream gene of Nrf2)
tBHQ	tert-butylhydroquinone
HO1	HO1 pathway/expression
PDE9	Phosphodiesterase-9
ORAC-FL	Oxygen Radical Absorbance Capacity (test)

## References

[1] Gadhave D.G., Sugandhi V.V., Jha S.K., Nangare S.N., Gupta G., Singh S.K., Dua K., Cho H., Hansbro P.M., Paudel K.R. (2024). Neurodegenerative disorders: Mechanisms of degeneration and therapeutic approaches with their clinical relevance. Ageing Res. Rev..

[2] Weller J., Budson A. (2018). Current understanding of Alzheimer’s disease diagnosis and treatment. F1000Research.

[3] Blaikie L., Kay G., Lin P.K.T. (2019). Current and emerging therapeutic targets of alzheimer’s disease for the design of multi-target directed ligands. MedChemComm.

[4] WHO (2022). A Blueprint for Dementia Research. https://www.who.int/publications/i/item/9789240058248.

[5] Wimo A., Ali G.-C., Guerchet M., Prince M., Prina M., Wu Y.-T. World Alzheimer Report 2015 The Global Impact of Dementia an Analysis of Prevalence, Incidence, Cost and Trends. www.alz.co.uk/worldreport2015corrections.

[6] Safiri S., Jolfayi A.G., Fazlollahi A., Morsali S., Sarkesh A., Sorkhabi A.D., Golabi B., Aletaha R., Asghari K.M., Hamidi S. (2024). Alzheimer’s disease: A comprehensive review of epidemiology, risk factors, symptoms diagnosis, management, caregiving, advanced treatments and associated challenges. Front. Med..

[7] Armstrong R.A. (2019). Risk factors for Alzheimer’s disease. Folia Neuropathol..

[8] Dugger B.N., Dickson D.W. (2017). Pathology of neurodegenerative diseases. Cold Spring Harb. Perspect. Biol..

[9] Albert M.S., DeKosky S.T., Dickson D., Dubois B., Feldman H.H., Fox N.C., Gamst A., Holtzman D.M., Jagust W.J., Petersen R.C. (2011). The diagnosis of mild cognitive impairment due to Alzheimer’s disease: Recommendations from the National Institute on Aging-Alzheimer’s Association workgroups on diagnostic guidelines for Alzheimer’s disease. Alzheimer’s Dement..

[10] Jack C.R., Andrews J.S., Beach T.G., Buracchio T., Dunn B., Graf A., Hansson O., Ho C., Jagust W., McDade E. (2024). Revised criteria for diagnosis and staging of Alzheimer’s disease: Alzheimer’s Association Workgroup. Alzheimer’s Dement..

[11] Shawkatova I., Javor J. (2025). Alzheimer’s Disease: Recent Developments in Pathogenesis, Diagnosis, and Therapy. Life.

[12] Francis P.T., Palmer A.M., Snape M., Wilcock G.K. (1999). The cholinergic hypothesis of Alzheimer’s disease: A review of progress. J. Neurol. Neurosurg. Psychiatry.

[13] Koelsch G. (2017). BACE1 Function and inhibition: Implications of intervention in the amyloid pathway of Alzheimer’s disease pathology. Molecules.

[14] Chen G.F., Xu T.H., Yan Y., Zhou Y.R., Jiang Y., Melcher K., Xu H.E. (2017). Amyloid beta: Structure, biology and structure-based therapeutic development. Acta Pharmacol. Sin..

[15] Tamagno E., Guglielmotto M., Vasciaveo V., Tabaton M. (2021). Oxidative Stress and Beta Amyloid in Alzheimer’s Disease. Which Comes First: The Chicken or the Egg?. Antioxidants.

[16] Tönnies E., Trushina E. (2017). Oxidative Stress, Synaptic Dysfunction, and Alzheimer’s Disease. J. Alzheimer’s Dis..

[17] Chen Z., Zhong C. (2014). Oxidative stress in Alzheimer’s disease. Neurosci. Bull..

[18] Tiwari S., Atluri V., Kaushik A., Yndart A., Nair M. (2019). Alzheimer’s disease: Pathogenesis, diagnostics, and therapeutics. Int. J. Nanomed..

[19] Lue L.F., Brachova L., Civin W.H., Rogers J. (1996). Inflammation, A beta deposition, and neurofibrillary tangle formation as correlates of Alzheimer’s disease neurodegeneration. J. Neuropathol. Exp. Neurol..

[20] Soares C., Ros L.U.D., da Rocha A.S., Machado L.S., Bellaver B., Zimmer E.R. (2022). The glutamatergic system in Alzheimer’s disease: A systematic review with meta-analysis. Alzheimer’s Dement..

[21] Nasb M., Tao W., Chen N. (2024). Alzheimer’s Disease Puzzle: Delving into Pathogenesis Hypotheses. Aging Dis..

[22] Sousa J.A., Bernardes C., Bernardo-Castro S., Lino M., Albino I., Ferreira L., Brás J., Guerreiro R., Tábuas-Pereira M., Baldeiras I. (2023). Reconsidering the role of blood-brain barrier in Alzheimer’s disease: From delivery to target. Front. Aging Neurosci..

[23] Behl T., Kaur D., Sehgal A., Singh S., Sharma N., Zengin G., Andronie-Cioara F.L., Toma M.M., Bungau S., Bumbu A.G. (2021). Role of Monoamine Oxidase Activity in Alzheimer’s Disease: An Insight into the Therapeutic Potential of Inhibitors. Molecules.

[24] Numakawa T., Kajihara R. (2023). Neurotrophins and Other Growth Factors in the Pathogenesis of Alzheimer’s Disease. Life.

[25] Cummings J. (2021). New approaches to symptomatic treatments for Alzheimer’s disease. Mol. Neurodegener..

[26] Atri A. (2019). The Alzheimer’s Disease Clinical Spectrum: Diagnosis and Management. Med. Clin..

[27] Cheong S.L., Tiew J.K., Fong Y.H., Leong H.W., Chan Y.M., Chan Z.L., Kong E.W.J. (2022). Current Pharmacotherapy and Multi-Target Approaches for Alzheimer’s Disease. Pharmaceuticals.

[28] Bubley A., Erofeev A., Gorelkin P., Beloglazkina E., Majouga A., Krasnovskaya O. (2023). Tacrine-Based Hybrids: Past, Present, and Future. Int. J. Mol. Sci..

[29] Silva M.A., Kiametis A.S., Treptow W. (2020). Donepezil Inhibits Acetylcholinesterase via Multiple Binding Modes at Room Temperature. J. Chem. Inf. Model..

[30] Kandiah N., Pai M.-C., Senanarong V., Looi I., Ampil E., Park K.W., Karanam A.K., Christopher S. (2017). Rivastigmine: The advantages of dual inhibition of acetylcholinesterase and butyrylcholinesterase and its role in subcortical vascular dementia and Parkinson’s disease dementia. Clin. Interv. Aging.

[31] Olivares D., Deshpande V.K., Shi Y., Lahiri D.K., Greig N.H., Rogers J.T., Huang X. (2012). N-Methyl D-Aspartate (NMDA) Receptor Antagonists and Memantine Treatment for Alzheimer’s Disease, Vascular Dementia and Parkinson’s Disease. Curr. Alzheimer Res..

[32] Hardy J. (2025). Alzheimer’s Disease: Treatment Challenges for the Future. J. Neurochem..

[33] Papaliagkas V. (2025). Anti-Amyloid Therapies for Alzheimer’s Disease: Progress, Pitfalls, and the Path Ahead. Int. J. Mol. Sci..

[34] Cummings J.L., Tong G., Ballard C. (2019). Treatment Combinations for Alzheimer’s Disease: Current and Future Pharmacotherapy Options. J. Alzheimer’s Dis..

[35] Zimmermann G.R., Lehár J., Keith C.T. (2007). Multi-target therapeutics: When the whole is greater than the sum of the parts. Drug Discov. Today.

[36] Tomaszewski S., Gauthier S., Wimo A., Rosa-Neto P. (2016). Combination Therapy of Anti-Tau and Anti-Amyloid Drugs for Disease Modification in Early-stage Alzheimer’s Disease: Socio-economic Considerations Modeled on Treatments for Tuberculosis, HIV/AIDS and Breast Cancer. J. Prev. Alzheimer’s Dis..

[37] Proschak E., Stark H., Merk D. (2018). Polypharmacology by Design: A Medicinal Chemist’s Perspective on Multitargeting Compounds. J. Med. Chem..

[38] Morphy R., Kay C., Rankovic Z. (2004). From magic bullets to designed multiple ligands. Drug Discov. Today.

[39] Bansal Y., Silakari O. (2014). Multifunctional compounds: Smart molecules for multifactorial diseases. Eur. J. Med. Chem..

[40] Gontijo V.S., Viegas F.P.D., Ortiz C.J.C., Silva M.d.F., Damasio C.M., Rosa M.C., Campos T.G., Couto D.S., Dias K.S.T., Viegas C. (2020). Molecular Hybridization as a Tool in the Design of Multi-target Directed Drug Candidates for Neurodegenerative Diseases. Curr. Neuropharmacol..

[41] Talevi A. (2015). Multi-target pharmacology: Possibilities and limitations of the ‘skeleton key approach’ from a medicinal chemist perspective. Front. Pharmacol..

[42] Zhou J., Jiang X., He S., Jiang H., Feng F., Liu W., Qu W., Sun H. (2019). Rational Design of Multitarget-Directed Ligands: Strategies and Emerging Paradigms. J. Med. Chem..

[43] Li X., Li X., Liu F., Li S., Shi D. (2021). Rational Multitargeted Drug Design Strategy from the Perspective of a Medicinal Chemist. J. Med. Chem..

[44] Morphy R., Rankovic Z. (2010). Medicinal Chemistry Approaches for Multitarget Drugs. Burger’s Medicinal Chemistry and Drug Discovery.

[45] Morphy R., Rankovic Z. (2009). Designing multiple ligands—Medicinal chemistry strategies and challenges. Curr. Pharm. Des..

[46] Morphy R., Rankovic Z. (2005). Designed multiple ligands. An emerging drug discovery paradigm. J. Med. Chem..

[47] Hopkins A.L., Mason J.S., Overington J.P. (2006). Can we rationally design promiscuous drugs?. Curr. Opin. Struct. Biol..

[48] Tavera-Mendoza L.E., Quach T.D., Dabbas B., Hudon J., Liao X., Palijan A., Gleason J.L., White J.H. (2008). Incorporation of histone deacetylase inhibition into the structure of a nuclear receptor agonist. Proc. Natl. Acad. Sci. USA.

[49] Sterling J., Herzig Y., Goren T., Finkelstein N., Lerner D., Goldenberg W., Miskolczi I., Molnar S., Rantal F., Tamas T. (2002). Novel dual inhibitors of AChE and MAO derived from hydroxy aminoindan and phenethylamine as potential treatment for Alzheimer’s disease. J. Med. Chem..

[50] Jiang X.-Y., Chen T.-K., Zhou J.-T., He S.-Y., Yang H.-Y., Chen Y., Qu W., Feng F., Sun H.-P. (2018). Dual GSK-3β/AChE Inhibitors as a New Strategy for Multitargeting Anti-Alzheimer’s Disease Drug Discovery. ACS Med. Chem. Lett..

[51] Jenwitheesuk E., Horst J.A., Rivas K.L., Van Voorhis W.C., Samudrala R. (2008). Novel paradigms for drug discovery: Computational multitarget screening. Trends Pharmacol. Sci..

[52] Ma X.H., Wang R., Tan C.Y., Jiang Y.Y., Lu T., Rao H.B., Li X.Y., Go M.L., Low B.C., Chen Y.Z. (2010). Virtual Screening of Selective Multitarget Kinase Inhibitors by Combinatorial Support Vector Machines. Mol. Pharm..

[53] Anighoro A., Bajorath J., Rastelli G. (2014). Polypharmacology: Challenges and Opportunities in Drug Discovery. J. Med. Chem..

[54] Simakov A., Chhor S., Ismaili L., Martin H. (2025). Nrf2 Activation and Antioxidant Properties of Chromone-Containing MTDLs for Alzheimer’s Disease Treatment. Molecules.

[55] Zhao L., Li B., Zheng L. (2024). Usnic Acid Derivatives as Multi-Target Anti-Alzheimer’s Disease Agents: Design, Synthesis, X-Ray Single Crystal Structure of Zn(II) Complex and Biological Activities. Chem. Biodivers..

[56] El-Mageed M.M.A., Ezzat M.A.F., Moussa S.A., Abdel-Aziz H.A., Elmasry G.F. (2024). Rational design, synthesis and computational studies of multi-targeted anti-Alzheimer’s agents integrating coumarin scaffold. Bioorganic Chem..

[57] Negi N., Ayyannan S.R., Tripathi R.K.P. (2025). Multi-targeted benzylpiperidine–isatin hybrids: Design, synthesis, biological and in silico evaluation as monoamine oxidases and acetylcholinesterase inhibitors for neurodegenerative disease therapies. J. Comput. Mol. Des..

[58] Shaaban A.E., Ali A.R., Ayyad S.N., Badria F.A. (2025). Unveiling the potential of novel natural product-based MTDLs that integrate cinnamamide scaffold as multifunctional agents for the treatment of Alzheimer’s disease. Bioorganic Chem..

[59] Dias I., Bon L., Banas A., Chavarria D., Borges F., Guerreiro-Oliveira C., Cardoso S.M., Sanna D., Garribba E., Chaves S. (2024). Exploiting the potential of rivastigmine-melatonin derivatives as multitarget metal-modulating drugs for neurodegenerative diseases. J. Inorg. Biochem..

[60] Asghar S., Mushtaq N., Ahmed A., Anwar L., Munawar R., Akhtar S. (2024). Potential of Tryptamine Derivatives as Multi-Target Directed Ligands for Alzheimer’s Disease: AChE, MAO-B, and COX-2 as Molecular Targets. Molecules.

[61] Wu X., Ze X., Qin S., Zhang B., Li X., Gong Q., Zhang H., Zhu Z., Xu J. (2024). Design, Synthesis, and Biological Evaluation of Novel Tetrahydroacridin Hybrids with Sulfur-Inserted Linkers as Potential Multitarget Agents for Alzheimer’s Disease. Molecules.

[62] Nagani A., Shah M., Patel S., Patel H., Parikh V., Patel A., Patel S., Patel K., Parmar H., Bhimani B. (2024). Unveiling piperazine-quinoline hybrids as potential multi-target directed anti-Alzheimer’s agents: Design, synthesis and biological evaluation. Mol. Divers..

[63] Żołek T., Purgatorio R., Kłopotowski Ł., Catto M., Ostrowska K. (2024). Coumarin Derivative Hybrids: Novel Dual Inhibitors Targeting Acetylcholinesterase and Monoamine Oxidases for Alzheimer’s Therapy. Int. J. Mol. Sci..

[64] Zhou Q., Wu X.-N., Luo W.-H., Huang Q.-H., Feng L.-L., Wu Y., Zhang C. (2025). Discovery of Effective Inhibitors Against Phosphodiesterase 9, a Potential Therapeutic Target of Alzheimer’s Disease with Antioxidant Capacities. Antioxidants.

[65] Polini B., Zallocco L., Gado F., Ferrisi R., Ricardi C., Zuccarini M., Carnicelli V., Manera C., Ronci M., Lucacchini A. (2024). A Proteomic Approach Identified TFEB as a Key Player in the Protective Action of Novel CB2R Bitopic Ligand FD22a against the Deleterious Effects Induced by β-Amyloid in Glial Cells. Cells.

[66] Bajad N.G., Jangra J., Ta G., Kumar A., Krishnamurthy S., Singh S.K. (2025). Discovery of pyrazoline analogs as multi-targeting cholinesterase, β-secretase and Aβ aggregation inhibitors through lead optimization strategy. Int. J. Biol. Macromol..

[67] Ahsan M.J., Ali A., Ali A., Thiriveedhi A., Bakht M.A., Yusuf M., Salahuddin, Afzal O., Altamimi A.S.A. (2022). Pyrazoline Containing Compounds as Therapeutic Targets for Neurodegenerative Disorders. ACS Omega.

[68] Kumar V., Jangid K., Kumar V., Kumar N., Mishra J., Arora T., Dwivedi A.R., Kumar P., Bhatti J.S., Parkash J. (2025). In vitro and in vivo Investigations of 4-Substituted 2-Phenylquinazoline derivatives as multipotent ligands for the treatment of Alzheimer’s disease. Bioorganic Chem..

[69] Kumar B., Kumar M., Dwivedi A.R., Kumar V. (2018). Synthesis, Biological Evaluation and Molecular Modeling Studies of Propargyl-Containing 2,4,6-Trisubstituted Pyrimidine Derivatives as Potential Anti-Parkinson Agents. ChemMedChem.

[70] Pachón-Angona I., Bernard P.J., Simakov A., Maj M., Jozwiak K., Novotna A., Lemke C., Gütschow M., Martin H., Oset-Gasque M.-J. (2024). Design and Synthesis of Multi-Functional Ligands through Hantzsch Reaction: Targeting Ca2+ Channels, Activating Nrf2 and Possessing Cathepsin S Inhibitory, and Antioxidant Properties. Pharmaceutics.

[71] Pinheiro P.d.S.M., de Chirico F., Loi M., Trazzi S., Ciani E., Rodrigues D.A., Alves M.A., Lima L.M., Milelli A., Monti B. (2025). Design, synthesis and pharmacological evaluation of multitarget GPR40 agonists/HDAC6 inhibitors for Alzheimer’s disease. Eur. J. Med. Chem..

[72] Said M.F., Wadie W., El-Haleim E.A.A., El Shiekh R.A., El-Zoheiry H.H. (2025). Probing new 3-hydrazinyl indole phenacetamide derivatives as multitarget anti-Alzheimer: Synthesis, in vivo, in vitro, and in silico studies. Eur. J. Med. Chem..

[73] Vitale R.M., Morace A.M., D’Errico A., Ricciardi F., Fusco A., Boccella S., Guida F., Nasso R., Rading S., Karsak M. (2024). Identification of Cannabidiolic and Cannabigerolic Acids as MTDL AChE, BuChE, and BACE-1 Inhibitors Against Alzheimer’s Disease by In Silico, In Vitro, and In Vivo Studies. Phytother. Res..

[74] D’ANiello E., Fellous T., Iannotti F.A., Gentile A., Allarà M., Balestrieri F., Gray R., Amodeo P., Vitale R.M., Di Marzo V. (2019). Identification and characterization of phytocannabinoids as novel dual PPARα/γ agonists by a computational and in vitro experimental approach. Biochim. Biophys. Acta (BBA)—Gen. Subj..

